# Artificial intelligence and computational prediction models for risk stratification, treatment response, and outcomes in colorectal cancer: a narrative review

**DOI:** 10.3389/fonc.2026.1944198

**Published:** 2026-09-22

**Authors:** Wenneng Liu, Dan Zhang, Chun Dang, Yaoheng Lu, Yinghan Song

**Affiliations:** 1 Department of General Surgery, Chengdu Integrated Traditional Chinese Medicine and Western Medicine Hospital, Chengdu, China; 2 Department of Periodical Press, West China Hospital, Sichuan University, Chengdu, China; 3 Department of Day Surgery Center, West China Hospital, Sichuan University, Chengdu, China

**Keywords:** artificial intelligence, clinical translation, colorectal cancer, digital pathology, radiomics, treatment response

## Abstract

**Purpose:**

To critically evaluate, within a decision-centred framework, the clinical readiness of artificial-intelligence (AI) and computational prediction models used for risk stratification, treatment-response assessment, and outcome prediction in colorectal cancer (CRC), while distinguishing tumour site, intended clinical decision, assessment time point, and validation level, and identifying the gap between predictive performance and clinical impact.

**Methods:**

PubMed/MEDLINE and Web of Science were searched for English-language studies published from January 2010 through 31 May 2026; ScienceDirect, Google Scholar, reference lists, and forward citation tracking were used as supplementary sources. Two reviewers independently screened studies and extracted design, endpoint, data modality, unit of analysis, validation, calibration, decision analysis, workflow evaluation, accessibility, and clinical-impact evidence. Study-level methodological features were assessed separately from task-level translational readiness.

**Results:**

Comparatively stronger evidence was found for computer-aided endoscopic assessment of invasion depth, MRI-based lymph-node prediction in rectal cancer, H&E whole-slide-image pre-screening for MSI/MMR status, and selected externally tested models for pathological complete response. Evidence remained early-stage for CT-based nodal staging in colon cancer, tumour mutational burden prediction, neoadjuvant immunotherapy-response prediction, synchronous distant-metastasis assessment, recurrence, survival, and most perioperative-risk models. Prospective validation was uncommon and was not equivalent to demonstration of clinical benefit.

**Conclusion:**

A decision-centred synthesis indicates that CRC AI is most mature as site-specific and task-specific reader assistance or triage rather than autonomous decision-making. Clinical translation requires representative external validation, calibration, predefined action thresholds, model availability, workflow testing, safety monitoring, and prospective evidence that model-guided care improves decisions or outcomes.

## 1 Introduction

Colorectal cancer (CRC) remains one of the most common malignancies worldwide and is a leading cause of cancer-related death. Its global burden is expected to increase further, strengthening the need for more accurate and clinically useful tools for staging, treatment selection, response assessment and prognostic evaluation (1). Contemporary CRC management increasingly depends not only on identifying a tumour, but also on estimating invasion depth, nodal and distant metastatic status, molecular phenotype, treatment sensitivity, perioperative risk, recurrence and survival. These estimates influence decisions that range from endoscopic resection versus surgery to neoadjuvant treatment, organ preservation, molecular testing and postoperative surveillance.

The relevant clinical questions differ substantially between colon and rectal cancer. Contrast-enhanced computed tomography (CT) is central to systemic staging in colon cancer, whereas high-resolution pelvic magnetic resonance imaging (MRI), assessment of the mesorectal compartment and response to neoadjuvant therapy are integral to rectal-cancer management. A model developed for rectal MRI therefore cannot be assumed to apply to colon CT, and prediction of pathological complete response (pCR) before treatment is not equivalent to post-treatment assessment of clinical complete response (cCR). Likewise, synchronous-metastasis assessment at baseline is distinct from prediction of metachronous recurrence after curative-intent treatment. These distinctions are essential because apparently similar model endpoints may lead to different clinical actions (2, 3).

Artificial intelligence (AI) is used inconsistently in the CRC literature. In this review, AI refers to machine-learning and deep-learning methods that learn predictive patterns from data, including convolutional neural networks, transformer architectures, multiple-instance learning and natural-language processing. Radiomics is treated as a quantitative feature-engineering approach rather than as AI in itself. Radiomics studies were retained when extracted imaging features were incorporated into an explicit predictive model, but the modelling method is identified as radiomics-statistical, radiomics-machine-learning or radiomics-deep-learning as appropriate. Conventional multivariable regression models based only on routine clinical variables were not classified as AI and were included only when they served as comparators or formed part of an explicitly AI-enabled clinical pathway. This terminology is intended to avoid conflating high-dimensional feature extraction, conventional statistical prediction and autonomous AI (4).

AI and computational models may derive information from endoscopic images, radiological examinations, H&E-stained whole-slide images (WSIs), immunohistochemistry (IHC), molecular data, electronic health records and multimodal combinations. Their potential value lies in standardising interpretation, identifying patterns not evident on routine inspection and integrating complex variables. However, favourable discrimination in a retrospective dataset does not establish clinical utility. Transportability depends on the patient spectrum, scanner or staining protocol, unit of data splitting, endpoint definition and treatment context; safe clinical use additionally requires calibration, clinically relevant thresholds, workflow evaluation and evidence that model-guided decisions improve care (5).

A recent review by Elahi et al. synthesised radiomics-based models across imaging modalities for CRC diagnosis, staging, hepatic metastasis, prognosis and treatment response (6). Although that review also discussed general challenges related to reproducibility, standardisation and limited external validation, it was organised primarily by imaging modality and clinical application. In contrast, the present review uses the intended clinical decision as its fundamental unit of analysis. Each model is interpreted according to tumour site, clinical setting, assessment time point, available input, reference standard, target action and consequences of error, while study-level methodological support is assessed separately from task-level translational readiness. This framework prevents clinically non-equivalent endpoints from being compared solely by AUC and explicitly distinguishes colon from rectal applications, synchronous metastasis from metachronous recurrence, pretreatment pCR prediction from post-treatment pCR or cCR assessment, and H&E-based MSI/MMR pre-screening from IHC workflow automation. It therefore identifies not only where predictive signals are reproducible, but also where evidence remains insufficient for AI to support or alter clinical decision-making.

The framework provides additional scientific insight by linking study-level methodological support to task-level translational readiness. It distinguishes external or prospective validation of predictive performance from evidence that model-guided care changes decisions or outcomes, and evaluates calibration, action thresholds, decision analysis, reader or workflow effects, accessibility and prospective impact. This synthesis reveals that maturity is asymmetric across the CRC pathway: the strongest evidence is concentrated in reader assistance and triage, whereas treatment selection, organ-preservation decisions, surveillance allocation and most prognostic uses remain early-stage. Across all reviewed applications, AI should be treated as task-specific decision support and should not independently guide treatment unless prospective clinical-impact studies demonstrate net benefit, safety and accountable workflow integration.

Within this framework, this structured narrative review covers AI and computational prediction for: (1) optical assessment of already detected early colorectal lesions; (2) site-specific regional-node and baseline distant-metastasis assessment; (3) image-derived MSI/MMR and tumour-mutational-burden (TMB) estimation; (4) pre-treatment and post-treatment response assessment in rectal cancer; and (5) perioperative complications, recurrence and survival. Population-level polyp detection during screening colonoscopy, purely technical segmentation without a clinical endpoint, basic histological classification, drug discovery and models for non-CRC populations are outside the scope. Throughout the review, model performance is interpreted in relation to the clinical decision it is intended to support rather than by the area under the receiver-operating-characteristic curve alone.

## 2 Data sources, study selection and qualitative evidence framework

### 2.1 Review design and information sources

This article was designed as a structured narrative review rather than a systematic review or meta-analysis. Its preparation was informed by the Scale for the Assessment of Narrative Review Articles (SANRA), particularly the need to justify the review question, describe the literature search, present relevant endpoint data, reference key claims and interpret evidence cautiously (7).

PubMed/MEDLINE and the Web of Science Core Collection were searched for English-language publications from 1 January 2010 through 31 May 2026. The final database searches were run on 10 June 2026. ScienceDirect and Google Scholar were used as supplementary sources to identify recent studies, publications not retrieved in the primary databases and relevant cited or citing articles. Reference lists of eligible reviews, meta-analyses, guidelines and key original studies were hand-searched, and forward citation tracking was undertaken for selected landmark studies. The database-specific strategies, fields, filters, dates and supplementary-search screening limits are reported in **Supplementary Methods S1**.

The searches combined three prespecified concept blocks: (1) CRC, colon cancer or rectal cancer; (2) AI, machine learning, deep learning, radiomics, pathomics, digital pathology, WSI, natural-language processing, computer-aided diagnosis or multimodal modelling; and (3) the clinical task. The third block explicitly covered endoscopy, invasion depth, submucosal invasion, endoscopic treatment selection, regional-node and lateral-node metastasis, synchronous and metachronous distant metastasis, MSI/MMR, TMB, neoadjuvant treatment, pCR, cCR, organ preservation, postoperative complications, anastomotic leakage, surgical-site infection, postoperative ileus, venous thromboembolism, readmission, length of stay, mortality, survival and recurrence.

For each of seven task-specific ScienceDirect and Google Scholar searches, the first 100 results ranked by relevance were screened. The seven groups were: early-lesion characterisation; regional nodal assessment; baseline distant-metastasis assessment; MSI/MMR, TMB and immunotherapy-related prediction; neoadjuvant response and organ preservation; perioperative complications; and survival or recurrence. Duplicate publications and studies already identified through PubMed or Web of Science were removed.

### 2.2 Eligibility and terminology

Eligible original studies evaluated a clinically interpretable diagnostic-state, treatment-response or prognostic task in CRC using machine learning, deep learning, radiomics-based predictive modelling, pathomics, digital pathology, WSI analysis, natural-language processing or multimodal modelling. Systematic reviews, meta-analyses, reporting or appraisal guidance and clinical guidelines were included to contextualise primary evidence. When several reports addressed the same task, priority was given to studies with representative cohorts, clinically meaningful endpoints, clearly defined reference standards, patient-level data separation, independent or multicentre validation, reader-assistance evaluation, prospective testing, workflow implementation or clinical-impact assessment. Earlier studies were retained when they were methodologically or conceptually important.

Radiomics was not assumed to constitute AI. A radiomics study was eligible when quantitative image features were linked to a prespecified clinical endpoint through an explicit statistical or machine-learning model; the actual algorithm is reported rather than relabelling every radiomics pipeline as AI. Conventional logistic- or Cox-regression nomograms using only routine clinicopathological variables were not treated as AI and were retained only as comparators or components of an AI-enabled workflow. This boundary was applied consistently in the text and tables.

Studies were excluded if they concerned non-CRC malignancies; animal or laboratory models without a clinically interpretable patient-level task; population-level screening or polyp detection without characterisation of an already detected lesion; purely technical segmentation or image classification without a clinically relevant endpoint; non-peer-reviewed reports; insufficiently described conference abstracts; duplicate cohorts without additional information; or publications whose methods or results could not be verified.

### 2.3 Study selection and data extraction

For each prespecified clinical task group, the first 100 records returned under relevance-based ranking were reviewed. The searches covered PubMed/MEDLINE, Web of Science, ScienceDirect and Google Scholar. Additional potentially relevant publications were identified through backward and forward citation searching and targeted searches prompted by eligible studies and relevant methodological, reporting or regulatory issues.

The electronic searches were conducted by Chun Dang (C.D.) and independently checked by Yaoheng Lu (Y.L.). The relevance and eligibility of potentially suitable publications were assessed by the same two reviewers. Disagreements regarding study selection, data extraction or interpretation were resolved through discussion and consensus. The 49 primary-study reports presented in **Tables 1**–**6** were selected as representative evidence for the corresponding clinical tasks rather than as an exhaustive set of formally included studies. The complete reference list additionally contains previous reviews, clinical and regulatory guidelines, reporting standards, risk-of-bias frameworks and other methodological sources used to support interpretation of the evidence.

**Table 1:** Representative studies of artificial intelligence for endoscopic invasion-depth assessment and treatment selection in colorectal neoplasia.

Study	Clinical context	Prediction task; unit of analysis and splitting	Input and AI method	Sample/positive events	Validation design	Performance	Clinical interpretation and key limitations
Tamura et al., 2026 (19)	Both; T1 colorectal cancer	Lesion-level multimodal T1 CRC assessment; reader-assistance study; split unit NR.	WLI, magnifying endoscopy, endocytoscopy and EndoBRAIN-Plus CADx	n=66 lesions, including 18 T1b cancers (27.3%).	Single-centre reader-assistance evaluation; no geographically external validation.	AI-assisted diagnosis achieved accuracy 88.9% and specificity 93.1%; high-confidence readings increased from 40.2% (WLI) to 75.5% (AI). Trainee high-confidence accuracy with AI reached 94.5% versus 96.2% for experts.	May support adjunctive assessment, especially for reducing expertise-related gaps. No external validation or prospective resection-decision evaluation. Single-centre; no external validation; no prospective assessment of patient management or outcomes.
Lui et al., 2019 (20)	Colon; large colonic lesions	Lesion-level curative-resection classification; 567 images from 76 lesions; patient-level split NR.	Non-magnified NBI and WLI; deep-learning image classifier	76 lesions; event rate NR	Single-centre internal evaluation; no geographically external validation.	Overall accuracy 85.5% for predicting endoscopic curative resection. NBI outperformed WLI (accuracy 94.3% vs 76.0%; AUROC 0.934 vs 0.758). AI outperformed two junior endoscopists and was similar to senior performance.	May support adjunctive assessment. Stronger with NBI than WLI; supports probability of curative endoscopic resection but should not replace expert/pathological criteria. Single-centre image-based study; no prospective real-time workflow validation; external generalisability uncertain.
Okamoto et al., 2022 (21)	Both; colorectal lesions, site not always separately analysed	Lesion/image-level NICE/JNET classification; split unit NR.	NBI/ME-NBI; ResNet18; CADx-N and CADx-J for NICE/JNET classification	NR	Single-centre internal evaluation; no geographically external validation.	CADx-N accuracies for NICE types 1, 2 and 3 were 97.5%, 91.2% and 93.8%, respectively; performance was stable across magnification levels. CADx-J showed robust multi-class performance including high sensitivity/NPV for type 1 and high specificity for type 3.	May support optical classification and education, but indirect for deciding endoscopic resection because classification performance is not identical to treatment-indication validation. Single-centre retrospective image evaluation; no prospective clinical decision trial; lesion-site and operator effects require further study.
Luo et al., 2021 (22)	Both; early colorectal neoplasms	Lesion-level non-invasive/superficial versus deep invasion; independent lesion-level test set; patient-level independence NR.	Non-magnified white-light colonoscopy; attention-guided GoogLeNet-based CNN.	Training: 657 lesions/7,734 images; test: 156 lesions/1,634 images; deep-invasion event count NR.	Internal independent test set; no geographically external centre validation.	For non-invasive/superficial lesions: accuracy 91.1%, sensitivity 91.2%, specificity 91.0% and AUROC 0.970. For superficial versus deeply invasive early CRC: sensitivity 65.3% and AUROC 0.729, similar to experienced endoscopists.	May support identification of lesions suitable for endoscopic therapy, but sensitivity for deep invasion is limited; not sufficient as a stand-alone rule-out tool. Heterogeneous lesion types and imaging; modest sensitivity for deep invasion; prospective real-time validation still needed.
Tokunaga et al., 2021 (24)	Both; colorectal lesions	Lesion-level classification of endoscopically treatable versus surgically indicated lesions; image-level internal split; lesion/patient-level independence was not reported.	Non-magnified, non-stained white-light endoscopy; Single Shot MultiBox Detector-based CAD.	1,035 consecutive lesions/3,442 images: 105 LGDs, 377 HGDs, 107 CRCs with SM invasion <1,000 μm, 146 CRCs with SM invasion ≥1,000 μm and 300 advanced CRCs; 589 treatable and 446 non-treatable by the study definition. Development 2,751 images; test 691 images.	Single-centre retrospective internal image-level hold-out evaluation; lesion/patient-level separation was not reported; no external or real-time validation.	Sensitivity 96.7%, specificity 75.0%, PPV 90.2%, NPV 90.5%, AUROC 0.913 and accuracy 90.3% for treatability classification. Trainees achieved sensitivity, specificity and accuracy of 92.1%, 67.6% and 84.9%, respectively; the corresponding values for experts were 96.5%, 72.5% and 89.4%. The correct-classification rate for T1b CRC was 51.2%.	The overall result supports adjunctive white-light assessment, but the modest specificity, low T1b correct-classification rate, image-level split and absence of external/prospective real-time evaluation preclude autonomous treatment selection.
Nakajima et al., 2020 (23)	Both; Tis/T1 colorectal lesions	Lesion-level T1b identification; development in two hospitals and testing in a third hospital.	Non-magnified WLI; ResNet-50 CAD for T1b identification	Development: 313 sessile CRCs/1,839 images from two hospitals; geographically external test: 44 CRCs/78 images (23 Tis and 21 T1b).	Two-hospital development followed by a third-hospital geographically external still-image test; the test set contained no T1a lesion.	At the prespecified lesion-level threshold (>0.95), AUROC was 0.888; sensitivity 81%, specificity 87%, PPV 85%, NPV 83% and accuracy 84%. Expert accuracies were 93% and 91%; trainee accuracies were 64% and 80%. The reported 87% versus 61% comparison referred to specificity, not overall accuracy.	Geographic separation supports transportability beyond an internal split, but the small enriched test set, absence of T1a lesions, selected still images and lack of prospective real-time testing limit clinical use.
Song et al., 2020 (25)	Both; colorectal polyps/early lesions	Lesion-level histology/invasion-risk classification; split unit NR.	NBI; DenseNet-201; histology and invasion-risk classification	NR	Single-centre internal evaluation; no geographically external validation.	Accuracy 81.3%-82.4%, kappa 0.614-0.642, AUROCs 0.86-0.95; CAD significantly outperformed trainees and, with assistance, improved trainee performance toward expert level.	Indirect. Useful for optical histology and risk classification; less direct evidence for final endoscopic-resection eligibility. No external validation; not purely an invasion-depth/treatment-indication endpoint; limited evidence for real-world decision improvement.
Zorron et al., 2020 (26)	Both; colorectal lesions	Lesion-level optical classification; internal and external image sets; patient-level independence NR.	NBI and BLI; DenseNet; modified Sano classification	NR	Internal validation plus external image-set validation; patient-level independence was not clearly reported.	Internal AUC 0.943; external AUC 0.845 for NBI and 0.903 for BLI; performance was comparable with experts in classification tasks.	May support adjunctive use. External image validation supports classification reliability, but treatment decisions still require full clinical and pathological context. Image-based validation; no prospective real-time workflow; clinical thresholds for resection decisions require standardisation.
Ito et al., 2019 (27)	Both; early colorectal lesions	Lesion/image-level cT1b classification; 190 images from 41 cases; 3-fold cross-validation.	WLI; AlexNet/Caffe CNN for cT1b diagnosis	41 cases: 14 cTis, 14 cT1a and 13 cT1b; 190 white-light images.	Single retrospective dataset with internal 3-fold cross-validation; no external validation.	For cT1b diagnosis: AUC 0.871, sensitivity 67.5%, specificity 89.0%, accuracy 81.2%.	The reported specificity may support adjunctive identification of deep invasion, but moderate sensitivity means it cannot safely rule out T1b disease alone. No external validation; sensitivity too low for stand-alone exclusion of deep invasion; no prospective workflow assessment.
Tamai et al., 2017 (28)	Both; colorectal lesions	Lesion-level magnifying-NBI classification; 121 lesions.	ME-NBI; software-based CAD using Sano classification features	121 lesions; event rate NR	Single-centre pilot internal evaluation; no geographically external validation.	In 121 lesions, CAD-endoscopist concordance 90.9%; diagnostic accuracy 82.8%, sensitivity 83.9%, NPV 95.6% for classification including SM-deep lesions.	Pilot evidence may support adjunctive use. High NPV may help triage, but evidence is insufficient for definitive treatment selection. Small pilot study; older feature-engineered software; no external/prospective validation.

AUC/AUROC, area under the receiver operating characteristic curve; CAD, computer-aided diagnosis; CNN, convolutional neural network; CRC, colorectal cancer; HGD, high-grade dysplasia; LGD, low-grade dysplasia; NPV, negative predictive value; PPV, positive predictive value; SM, submucosal; WLE/WLI, white-light endoscopy/imaging. NR, not reported.

**Table 2:** Representative computational models for lymph-node metastasis prediction in colorectal cancer.

Study	Clinical context	Prediction task; unit of analysis and splitting	Modality and AI method	Sample/positive events	Validation design	Performance	Clinical interpretation and key limitations
Xia et al., 2024 (29)	Rectal cancer; T1-T4; regional LNM on MRI	Patient-level rectal LNM from tumour MRI; training/internal/external cohorts separated by patient and centre.	High-resolution T2WI and DWI; weakly supervised ResNet-based WISDOM model	Total n=1,014: training n=589 (LNM prevalence 39.0%), internal test n=146 (36.3%), external test cohort 1 n=117 (34.2%), and external test cohort 2 n=162 (45.0%). Exact cohort-specific LNM-positive counts were not explicitly reported.	Multicentre development with an internal test cohort and two geographically external test cohorts.	Binary N0 versus N+ AUC 0.81 (95% CI, 0.77–0.86); ternary N0/N1/N2 C-index 0.765 (95% CI, 0.727–0.802). WISDOM outperformed junior radiologists and was comparable with senior radiologists; AI assistance increased junior AUC to 0.80 and senior AUC to 0.88.	Multicentre external testing and reader-assistance evaluation support adjunctive rectal-MRI nodal assessment. Although calibration curves and decision-curve analysis were reported, quantitative calibration measures, fixed clinical action thresholds and prospective workflow-impact evaluation remain lacking.
Wan et al., 2023 (30)	Rectal cancer; stage T1-T2	Patient-level T1-T2 rectal LNM; tumour-based MRI; single-centre patient split.	Preoperative high-resolution T2WI MRI; 2D ResNet50 and 3D ResNet101	Total n=611; test cohort n=86, including 19 LNM-positive patients (22.1%).	Single-centre internal train/validation/test split.	Eight DL models achieved AUCs of 0.77–0.89 in the training and validation cohorts. The best 3D ResNet101 model achieved a test AUC of 0.79 (95% CI, 0.70–0.89) and outperformed pooled radiologist assessment (AUC, 0.54; 95% CI, 0.48–0.60; P<0.001).	May support research-level adjunctive staging in early rectal cancer; not validated broadly enough for stand-alone decisions. No external validation; single-centre; tumour-based MRI model rather than direct node-level pathology; limited to T1-T2 rectal cancer.
Kasai et al., 2022 (31)	Rectal cancer; lower rectal cancer; lateral lymph nodes	Side-level lateral-node metastasis; clustered observations within patients.	Preoperative clinicopathological and MRI variables; Prediction One (Sony Network Communications) machine-learning model.	Development: 267 patients/500 pelvic sides, including 46 LLNM-positive sides; subsequent temporal validation: 56 patients/107 sides, including 10 LLNM-positive sides.	Single-centre development with a subsequent temporal validation cohort; side-level observations were clustered within patients; no geographically external validation.	Validation AUROC was 0.903 (95% CI, 0.832–0.974), compared with 0.754 (95% CI, 0.578–0.930; P = .022) for the conventional diameter criterion. Accuracy, sensitivity and specificity were 80.4%, 90.0% and 79.4%, respectively. The model identified 4 of 5 LLNM-positive sides in which the largest node measured ≤4 mm.	Promising for side-specific LLNM risk stratification, but the estimate was based on only 10 LLNM-positive validation sides and had a wide confidence interval. This sparse-event result was treated as supportive evidence and was not used to increase task-level readiness. The single-centre temporal design and within-patient clustering further limit generalisability and treatment-decision use.
Ao et al., 2025 (32)	Rectal cancer; T1-T4; MRI-based LNM status	Patient-level rectal LNM; multicentre internal and geographically external validation.	Multiparametric MRI (DWI, T2WI, contrast-enhanced T1WI); ResNet-101 DL radiomics signature and nomogram	n=430 from two centres: training n=231, internal validation n=99 and external validation n=100. Source **Table 1** reports 189 LNM-positive cases (89/231, 53/99 and 47/100); these cohort-specific counts were used. The abstract reports 192/430, which could not be reconciled and was not used.	Centre 1 random 7:3 training/internal split; centre 2 geographically external validation.	Detailed source **Table 2**: DLRS AUC 0.97/0.99/0.95 (accuracy 0.96/0.97/0.93) and nomogram AUC 0.98/0.99/0.95 (accuracy 0.97/0.99/0.94) in training/internal/external cohorts; DCA favoured DLRS/nomogram. The source Results section mislabels the corresponding accuracy values as AUC values.	Supportive, hypothesis-generating retrospective evidence only. Although geographically external discrimination was high, the manual single-slice tumour input, high-dimensional feature selection, source-level reporting inconsistencies, absence of node-level analysis and lack of prospective workflow evaluation preclude decisive weight in the readiness assessment.
Li J. et al., 2021 (35)	Rectal cancer; MRI-based nodal status	Screenshot-level MRI classification (325 screenshots/129 patients); patient-level independence not clearly reported.	T2WI MRI screenshots; Inception-v3 deep transfer learning	129 patients/325 screenshots; LNM event rate NR	Single-centre internal validation with screenshot-level sampling; patient-level independence was not clearly reported.	Reported AUC 0.994 and accuracy 95.7%; sensitivity, specificity, PPV and NPV were approximately 95%; performance exceeded manual radiologist assessment.	Potentially useful but reliability is uncertain despite high performance, because the dataset and validation design may overestimate real-world accuracy. Small single-centre image/screenshot dataset; no independent external validation; likely risk of overfitting and spectrum bias.
Bedrikovetski et al., 2024 (34)	Colon cancer; preoperative CT nodal staging	Patient-level colon LNM prediction; patients were divided into training, internal-validation and retrospective held-out test cohorts, followed by evaluation in a prospectively accrued temporal test cohort; no geographically independent cohort was established.	CE and non-CE CT; ResNet-50 deep learning	Total n=1,201: training n=401, internal validation n=100, retrospective held-out test cohort 1 n=500 and prospectively accrued temporal test cohort 2 n=200; LNM event rate NR.	Ambispective evaluation: retrospective training, internal validation and held-out testing in cohort 1, followed by testing of the developed model in a prospectively accrued temporal cohort 2; geographically independent external validation was not established.	Validation AUROC 0.619 (95% CI, 0.507–0.731); retrospective held-out test AUROC 0.542 (95% CI, 0.489–0.595); prospectively accrued temporal test AUROC 0.486 (95% CI, 0.403–0.568), indicating near-chance discrimination.	No. Discrimination fell to near-chance levels in both the retrospective held-out and prospectively accrued temporal test cohorts, possibly reflecting the limited CT signal for nodal micrometastasis. The study provides evidence of temporal validation failure rather than geographically external validation; geographically independent validation, fixed clinical thresholds and prospective model-impact evidence remain absent.
Li et al., 2023 (33)	Both; T1 CRC assessed by CT and surgical pathology	Patient-level T1 CRC LNM; study population underwent CT and surgical resection; prior endoscopic resection was not required.	Contrast-enhanced CT morphology plus tumour location; logistic regression model	n=144; LNM 30/144 (20.83%).	Single retrospective cohort with 1,000-bootstrap internal validation; no centre-held-out external validation.	Prediction model showed good internal calibration/discrimination with reported C-index 0.89. Tumour location and CT morphologic features were associated with LNM.	May inform discussion of additional surgery, but the cohort was not restricted to patients after endoscopic resection. Small sample, internal validation only, and treatment thresholds require prospective testing.
Shi et al., 2026 (36)	Both; T1 CRC; clinicopathologic/endoscopic LNM risk	Patient-level T1 CRC LNM; 10-fold cross-validation; held-out test cohort.	Clinical, endoscopic and pathological variables; eight ML algorithms including RF, SVM, LR, MLP	n=210, including 51 LNM-positive patients (24.3%).	Single-centre 10-fold cross-validation with a held-out internal test cohort.	Random forest performed best with an AUC of 0.872 (95% CI, 0.8158–0.8953). Key predictors included submucosal infiltration area, proportion of invasive carcinoma, high-grade poorly differentiated clusters and serrated lesions.	May support T1 CRC LNM risk stratification and tailored post-endoscopic surgery decisions, but research-level only. Single-centre; no external validation; small testing cohort; requires prospective calibration and decision-curve analysis.
Zou et al., 2026 (37)	Both; advanced CRC T3/T4; WSI-based LNM risk	Case/slide/patch-level WSI MIL; Monte Carlo cross-validation; patient-level cases.	H&E WSIs; case-level multiple-instance learning with CONCH v1.5; clinical-feature integration	n=130; LNM event rate NR	Single-centre 5-fold Monte Carlo cross-validation.	Case-level CONCH v1.5 achieved mean AUC 0.899 versus slide-level AUC 0.814. Pathology + clinical SVM achieved mean AUC 0.904 compared with 0.584 for clinical-only.	May support pathology-based adjunctive assessment and interpretable feature-discovery tool; not ready for general clinical deployment. Small cohort; advanced CRC rather than early T1 decision context; no external validation; WSI workflow and reproducibility require testing.

ADC, apparent diffusion coefficient; CEA, carcinoembryonic antigen; CI, confidence interval; CRT, chemoradiotherapy; DCA, decision-curve analysis; DLRS, deep-learning radiomics score; DWI, diffusion-weighted imaging; LLN/LLNM, lateral lymph node/metastasis; LNM, lymph-node metastasis; MRI, magnetic resonance imaging; ROI, region of interest. NR, not reported.

**Table 3:** Representative studies of artificial intelligence and radiomics for synchronous distant-metastasis assessment in colorectal cancer.

Study	Clinical context	Prediction task	Unit of analysis and splitting	Modality and AI method	Sample/positive events	Validation design	Performance	Clinical interpretation and key limitations
Liu et al., 2020 (39)	Rectal cancer; primary rectal cancer; synchronous liver metastasis	Patient-level synchronous liver-metastasis risk	Patient-level; split by cohort; patient independence assumed.	T2WI MRI radiomics signature combined with CEA and CA19-9; radiomics nomogram	n=127, including 32 SLM events (25.2%).	Single-centre internal train/validation split; no geographically external validation.	The combined nomogram outperformed the radiomics-only model. AUCs were 0.918 and 0.944 in the training and validation cohorts, respectively; validation sensitivity 95.83%, specificity 88.89%, accuracy 90.80%.	May support adjunctive use for preoperative SLM risk stratification in primary rectal cancer; should not replace standard staging or MDT assessment. Small single-centre cohort; no independent external validation; restricted to liver metastasis and primary rectal cancer.
Mou et al., 2024 (40)	Rectal cancer; synchronous liver metastasis	Patient-level synchronous liver-metastasis risk	Patient-level; split by cohort.	Endoscopic rectal ultrasound radiomics plus clinical variables; radiomics-clinical ML model	n=239, including 34 SLM events (14.2%); validation cohort included 10 positive events.	Single-centre internal train/validation split; no geographically external validation.	The radiomics-clinical model achieved the best performance, with AUCs of 0.920 in training and 0.855 in validation, outperforming clinical-only and radiomics-only models.	The reported internal validation supports further evaluation as a research-level adjunct for rectal-cancer synchronous liver-metastasis prediction. Single-centre retrospective design; no geographically external validation; ultrasound acquisition and feature reproducibility require further testing.
Hu et al., 2019 (42)	CRC; indeterminate pulmonary nodules; lung metastasis prediction	Characterisation of an already detected indeterminate pulmonary nodule	Lesion-characterisation task; pulmonary-nodule/patient clustering NR.	Chest CT radiomics plus clinical variables; clinical-radiomics nomogram	n=194, including 93 metastatic pulmonary nodules (47.9%).	Single-centre internal train/validation split; no geographically external validation.	The nomogram achieved AUCs of 0.929 and 0.922 in the training and validation cohorts, respectively, with favourable calibration.	Internal validation suggests potential utility for differentiating metastatic from non-metastatic IPNs, but still adjunctive and context-specific. Single-centre cohort; no independent external validation; applies to patients with IPNs rather than general CRC metastasis screening.
Jin et al., 2023 (43)	CRC; pelvic bone metastasis	Detection/classification of pelvic bone metastasis on MRI	Lesion-detection/classification task; cross-validation unit and patient independence NR.	MRI with deep-learning-assisted pelvic-bone segmentation; GLCM radiomics and ML classifiers	n=614, including 53 pelvic bone-metastasis events (8.6%).	Single-centre retrospective random 7:3 split into a training set (n=429) and an internal validation set (n=185); ten-fold cross-validation was used for model/feature selection; no geographically external validation.	The random forest model achieved AUCs of 0.926 in training and 0.919 in internal validation, outperforming other evaluated models whose AUCs ranged from 0.716 to 0.912.	May support pelvic bone metastasis detection or risk warning within the pelvic MRI setting, but clinical use remains research-level. Internal validation only; metastatic-site-specific task; real-world radiology workflow and external generalisability not established.
Jiang et al., 2026 (41)	Rectal cancer; synchronous distant metastasis	Baseline classification of synchronous distant metastasis already present at staging, rather than prediction of future metachronous metastasis.	Patient-level; single-centre train/test split.	Multiparametric MRI radiomics nomogram using T2WI and DWI plus clinical variables	n=169; training n=134 and internal test n=35; SDM 82/169 (48.5%).	Single-centre retrospective stratified 8:2 internal hold-out split; no geographically external validation.	AUC 0.93 (95% CI, 0.89–0.96) in training and 0.94 (95% CI, 0.79–0.97) in testing; sensitivity 0.85–0.88 and specificity 0.86–0.89. Calibration plots showed agreement, and DCA favoured the clinical-radiomics nomogram over standalone models.	High internal discrimination supports further evaluation as a staging adjunct, but the 35-patient test set, selected high SDM prevalence, single-centre design and absence of external/prospective impact testing preclude clinical deployment.

CEA, carcinoembryonic antigen; CI, confidence interval; CRC, colorectal cancer; CT, computed tomography; DCA, decision-curve analysis; DWI, diffusion-weighted imaging; IPN, indeterminate pulmonary nodule; MRI, magnetic resonance imaging; SDM/SLM, synchronous distant/liver metastasis. Patient-level risk, lesion characterisation and site-specific detection are non-equivalent tasks. NR, not reported.

**Table 4:** Representative pretreatment models for predicting pathological response to neoadjuvant therapy in rectal cancer.

Study	Clinical context	Neoadjuvant regimen and prediction time point	Prediction task; unit and splitting	Input and AI method	Sample/pCR events	Validation design	Reference standard	Performance	Clinical interpretation and key limitations
Boldrini et al., 2022 (61)	Rectal cancer; LARC; pCR after nCRT	Conventional nCRT; baseline MRI before treatment	Patient-level response prediction; baseline inputs; data split at patient level unless NR.	T2WI MRI radiomics model using clinical and radiomics features; LR	Original European model n=161; intercontinental external validation n=59, including pCR 10/59 (16.9%).	Geographically external validation (training n=161; external validation n=59).	Surgical pathology pCR	External AUC 0.83 (95% CI, 0.71-0.96), compared with 0.75 in the original validation. At the predefined cut-off: accuracy 0.65, specificity 0.64, sensitivity 0.70, NPV 0.91 and PPV 0.28.	External validation supports identifying patients unlikely to achieve pCR (ruling out pCR), but the low PPV prevents confirmation of pCR or treatment de-escalation. pCR prediction alone cannot justify watch-and-wait.
Feng et al., 2022 (62)	Rectal cancer; LARC; pCR after nCRT	Conventional nCRT; pretreatment mpMRI and biopsy WSI	Patient-level response prediction; baseline inputs; data split at patient level unless NR.	RAPIDS radiopathomics model integrating pretreatment mpMRI radiomics, pathomics nuclear features and pathomics microenvironment features; SVM	Development n=303 (pCR rate 28%); external cohorts n=480 and n=150 (22% and 20%); prospective cohort n=100 (23%).	Multicentre retrospective geographically external validation plus prospective observational validation.	Surgical pathology pCR	AUC was 0.868 (95% CI, 0.825–0.912) in training, 0.860 (0.828–0.892) and 0.872 (0.810–0.934) in the two retrospective external cohorts, and 0.812 (0.717–0.907) in the prospective observational cohort. RAPIDS outperformed the single-modality models, but this predictive-performance validation did not establish model-guided clinical benefit.	This study provides comparatively stronger pCR-prediction evidence because of multimodal data, large cohorts, external validation and prospective testing; still adjunctive. Observational validation; requires imaging-pathology integration and standardised workflows; no trial proving improved treatment selection or organ preservation.
Yardimci et al., 2023 (64)	Rectal cancer; LARC; response to nCRT	Conventional nCRT; baseline sagittal T2WI	Patient-level response prediction; baseline inputs; data split at patient level unless NR.	Pre-treatment sagittal T2WI MRI radiomics with multiple ML algorithms	n=76; pCR events/rate NR	Single-centre 10-fold cross-validation.	Pathological response after surgery	The best T2WI radiomics-only model achieved AUCs of 0.753 in validation and 0.705 in testing; DCA supported potential clinical utility versus clinical-only models.	Reported discrimination was limited to moderate; useful as exploratory evidence rather than clinical decision support. Small single-centre cohort; no external validation; only modest test AUC; algorithm details incompletely reported in the abstract.
Mao et al., 2022 (65)	Rectal cancer; LARC; response to nCRT	Conventional nCRT; baseline contrast-enhanced CT	Patient-level response prediction; baseline inputs; data split at patient level unless NR.	Contrast-enhanced CT radiomics plus clinical variables including distance to anal verge, lymphocyte-to-monocyte ratio and CEA; LR	n=216; pCR 44/216 (20.4%).	Single-centre internal random split.	Pathological response after surgery	The combined model achieved AUCs of 0.926 and 0.872 in training and validation, outperforming radiomics-only and clinical-only models.	May support internal risk stratification for identifying responders, but reliability remains limited without external validation. Random-split validation only; CT radiomics harmonisation and generalisability uncertain; clinical treatment-impact thresholds not established.
Tochigi et al., 2022 (66)	Rectal cancer; LARC; pCR after nCRT	Conventional nCRT; baseline contrast-enhanced CT	Patient-level response prediction; baseline inputs; data split at patient level unless NR.	Pretreatment contrast-enhanced CT texture/fractal-dimension analysis; LR	n=215; pCR 45/215 (20.9%).	Single-centre internal random split.	Surgical pathology pCR	Training AUC was 0.76. In validation, accuracy was 82%, specificity 89% and sensitivity 60%.	Clinical utility is limited because sensitivity was modest; may support hypothesis generation but is insufficient for ruling out non-pCR. No external validation; moderate sensitivity; limited feature set; prospective utility for treatment decisions not shown.
Shen et al., 2020 (67)	Rectal cancer; response after nCRT	Conventional nCRT; baseline 18F-FDG PET/CT	Patient-level response prediction; baseline inputs; data split at patient level unless NR.	Baseline 18F-FDG PET/CT radiomics features; random forest classifier	n=169; pCR 22/169 (13.0%).	Single-centre internal development using bootstrap-aggregated random forests, followed by five-fold cross-validation; no independent external validation.	Surgical pathology pCR	The selected RF achieved an apparent AUC of 0.941. Apparent sensitivity, specificity, PPV, NPV and accuracy were 81.8%, 97.3%, 81.8%, 97.3% and 95.3%, respectively. Mean five-fold cross-validation values were 0.74, 0.92, 0.67, 0.96 and 0.89, respectively; cross-validated AUC was not reported.	High internal performance was reported, especially specificity and NPV, but clinical reliability is uncertain without external validation. No cross-validated AUC was reported; PET/CT availability and cost; no AUC reported; risk of overfitting and spectrum bias.
Wu et al., 2025 (63)	Rectal cancer; LARC; pCR after nCRT	Conventional nCRT; baseline MRI	Patient-level response prediction; baseline inputs; data split at patient level unless NR.	T2WI MRI sub-regional radiomics integrated with 3D ResNet50 deep-learning signatures; SVM	Total n=768: training n=401, including 127 pCR events (31.7%); two geographically external cohorts n=209 and n=158, with 104 pCR events in aggregate across the external cohorts (104/367, 28.3%); overall 231/768 patients achieved pCR (30.1%).	Development cohort with two geographically external test cohorts.	Surgical pathology pCR	SRADL achieved AUCs of 0.925 (95% CI, 0.894–0.948), 0.915 (95% CI, 0.869–0.949), and 0.902 (95% CI, 0.846–0.945) in the training, external test 1, and external test 2 cohorts, respectively. Predicted pCR status was associated with survival stratification.	External validation supports an externally validated pretreatment adjunct for pCR prediction, but still not sufficient for autonomous organ-preservation decisions. Retrospective modelling; segmentation and harmonisation requirements; prospective treatment-decision and watch-and-wait impact not tested.
Zhang et al., 2024 (68)	Rectal cancer; LARC; pCR and prognosis after nCRT	Conventional nCRT; pretreatment biopsy H&E WSI	Patient-level response prediction; baseline inputs; data split at patient level unless NR.	H&E-stained whole-slide-image pathomics features extracted with CellProfiler; multiple ML classifiers including XGBoost and stacking	Development cohort: n=244, including 47 pCR events (19.3%); geographically external validation cohort: n=50, including 8 pCR events (16.0%); total 55/294 pCR events (18.7%).	Single-centre development with geographically external institutional validation.	Surgical pathology pCR	The XGBoost model achieved a development AUC of 1.000 (95% CI, 0.999–1.000). In the external cohort, the detailed Results reported an AUC of 0.908 (95% CI, 0.815–1.000), whereas the abstract reported 0.950. External DFS and OS comparisons were not statistically significant (P = .072 and P = .224 in the detailed Results; the abstract reported P = .074 and P = .224). The XGB score was an independent predictor of DFS only in the internal multivariable analysis (HR, 0.128; 95% CI, 0.026–0.636; P = .012).	May support pathomics-based adjunctive assessment, but the perfect development AUC, the small external cohort with only eight pCR events and the source-level discrepancy between the abstract and detailed Results indicate a substantial risk of overfitting. WSI preprocessing and pathology-workflow reproducibility require further validation.

AUC, area under the receiver operating characteristic curve; CEA, carcinoembryonic antigen; CI, confidence interval; CT, computed tomography; DCA, decision-curve analysis; LARC, locally advanced rectal cancer; nCRT, neoadjuvant chemoradiotherapy; NPV, negative predictive value; pCR, pathological complete response; PPV, positive predictive value; WSI, whole-slide image. Only predictor variables available before treatment are included. NR, not reported.

**Table 5:** Representative post-treatment and longitudinal models for response assessment and organ-preservation support in rectal cancer.

Study	Clinical context	Regimen and assessment time point	Prediction task; unit and splitting	Input and AI method	Sample/positive events	Validation design	Reference standard	Performance	Clinical interpretation and key limitations
Horvat et al., 2018 (71)	Rectal cancer after neoadjuvant therapy	Post-treatment restaging MRI; regimen details heterogeneous/NR	Residual tumour versus pCR; patient-level split NR	Post-treatment T2WI MRI radiomics	n=114; pCR 21/114 (18.4%).	Single-centre development with internal validation.	Surgical pathology	AUC 0.93 (95% CI, 0.87-0.96); sensitivity 100%, specificity 91%, PPV 72% and NPV 100%.	May support residual-disease reader assessment, but pCR on surgical pathology is not equivalent to cCR for watch-and-wait.
Horvat et al., 2022 (125)	Rectal cancer after neoadjuvant therapy	Post-treatment restaging MRI	pCR prediction; internal development and geographically external validation	T2-weighted MRI radiomics combined with qualitative radiologist assessment	Internal development cohort n=114, including 21 pCR events (18.4%); geographically external cohort n=50, including 8 pCR events (16.0%).	Retrospective internal development with geographically external validation at an independent Brazilian centre.	Surgical pathology	External Model B AUC 0.83 (95% CI, 0.70-0.97); combined models improved inter-reader agreement (κ=0.82 vs 0.25) and slightly improved junior-radiologist specificity, PPV and NPV.	Supports external reader assistance for pCR assessment, but pathology-based pCR does not validate cCR selection or watch-and-wait safety.
Haak et al., 2022 (78)	Rectal cancer after chemoradiation	Post-treatment endoscopy	Endoscopic response classification; patient-level, image clustering within patients	Deep learning on post-treatment endoscopic images	n=226; complete response 109/226 (48.2%), non-complete response 117/226.	Single-centre internal validation; no geographically external centre/platform validation.	Clinical/endoscopic response with pathology/follow-up as reported	Combined-model AUCs ranged from 0.76 to 0.83; the best-performing model achieved an AUC of 0.83 (95% CI, 0.80–0.86), accuracy of 0.75 (95% CI, 0.72–0.79), sensitivity of 0.77 (95% CI, 0.73–0.80), specificity of 0.75 (95% CI, 0.72–0.79), PPV of 0.74 (95% CI, 0.70–0.77) and NPV of 0.77 (95% CI, 0.74–0.80). Calibration and DCA were not reported.	Adjunctive mucosal assessment only; false-negative residual disease is the key safety concern.
Williams et al., 2024 (79)	Rectal cancer treated with TNT	Post-TNT endoscopy; response and local-regrowth assessment	Endoscopic response/local-regrowth classification; patient-level split as reported	Convolutional neural network on endoscopic images	n=288; 2,717 images, of which 1,407 contained tumour; local-regrowth event count NR.	Internal test set plus a separate local-regrowth set; geographically external and multicentre independence were not established.	Composite clinical response and local-regrowth reference standards	AUC 0.98 in the primary test set and 0.92 in the local-regrowth set; all clinician groups outperformed the model for local-regrowth identification.	Useful as endoscopic reader support, but performance for local regrowth was inferior to clinicians; multicentre platform validation and safety evaluation are required.
Xia et al., 2025 (74)	Rectal cancer after nCRT	Mixed pre- and post-nCRT mpMRI	Automated pCR prediction; patient-level single-centre internal held-out test set.	MedSAM segmentation plus radiomics/XGBoost	Final cohort n=378. Source **Table 1** reports training n=264 (50 pCR and 214 non-pCR) and internal test n=114 (22 pCR and 92 non-pCR); the supplementary flowchart independently confirms the 264/114 split. The discordant counts of 112 and 144 elsewhere in the publication were treated as reporting errors and were not used.	Single-centre internal held-out testing using the reconciled 114-patient test cohort; no geographically external validation.	Surgical pathology pCR	Combined pre/post-treatment model: mean AUC 0.83 ± 0.04 (SD), mean accuracy 0.79 ± 0.04 (SD), mean sensitivity 0.68 ± 0.04 (SD), and mean specificity 0.82 ± 0.04 (SD); mean post-treatment DWI Dice coefficient 0.46 ± 0.15 (SD).	Mixed pre/post-treatment workflow. Discordant sample counts elsewhere in the source were treated as reporting errors; low post-treatment DWI segmentation performance and the absence of geographically external validation limit reliability and generalisability.
Chen et al., 2025 (75)	Rectal cancer after nCRT	Longitudinal tumour-marker changes before and after nCRT	Post-treatment pCR risk prediction; patient-level external cohorts	Dynamic tumour-marker score with XGBoost and SHAP	Development-centre cohort n=892: training n=624, including 141 pCR events (22.6%), and internal-validation n=268, including 61 pCR events (22.8%); external cohort 1 n=228, including 52 pCR events (22.8%), and external cohort 2 n=196, including 44 pCR events (22.4%); total n=1,316, including 298 pCR events (22.6%).	Development cohort with two geographically external validation cohorts.	Surgical pathology pCR	External AUCs 0.80 and 0.82; AUPRCs 0.519 and 0.593	Uses post-treatment information and may support restaging, but does not establish cCR or safe omission of surgery.
Cheng et al., 2021 (73)	Rectal cancer; LARC after nCRT	Baseline mpMRI radiomics plus post-treatment tumour dimensions	Hybrid/longitudinal pCR prediction; patient-level internal development/validation	Baseline T1WI/T2WI/T2FS radiomics; final pCR nomogram included post-treatment tumour length and thickness; LR	n=193; training n=128 and internal validation n=65; pCR event count NR.	Single-centre retrospective internal hold-out validation (training n=128; validation n=65); no geographically external validation.	Surgical pathology pCR	AUC 0.959 (95% CI, 0.927–0.991; sensitivity 1.000, specificity 0.833) in training and 0.912 (95% CI, 0.843–0.982; sensitivity 1.000, specificity 0.815) in validation; DCA suggested potential net benefit.	Not a pure pretreatment model because the final pCR nomogram includes post-treatment measurements; no external or clinical-impact validation.
Pang et al., 2026 (72)	Rectal cancer; LARC after nCRT	Post-treatment MRI; preoperative pCR assessment	Patient-level pCR prediction and prospective human-model interaction	Post-nCRT MRI radiomics/deep residual shrinkage network plus clinicopathological factors; SHAP interpretation	Retrospective training cohort n=575, including 126 pCR events (21.9%); retrospective test cohort n=248, including 54 pCR events (21.8%); prospective multicentre cohort n=207, including 59 pCR events (28.5%); total n=1,030, including 239 pCR events (23.2%).	Retrospective development/testing plus multicentre prospective validation and radiologist-assistance evaluation	Surgical pathology pCR	Prospective AUC 0.795 (95% CI, 0.723-0.859); AI assistance improved radiologist AUC and sensitivity, particularly for junior readers	Strengthens prospective reader-assistance evidence for post-treatment pCR assessment, but does not establish cCR, watch-and-wait safety or patient-outcome benefit.

AUC, area under the receiver operating characteristic curve; cCR, clinical complete response; CRT/nCRT, (neoadjuvant) chemoradiotherapy; DWI, diffusion-weighted imaging; MRI/mpMRI, (multiparametric) magnetic resonance imaging; pCR, pathological complete response; TNT, total neoadjuvant therapy. pCR confirmed at surgery is not equivalent to cCR used for non-operative management. NR, not reported.

**Table 6:** Representative studies of artificial intelligence and radiomics for recurrence and metachronous-metastasis prediction after curative-intent treatment.

Study	Clinical context	Recurrence task; unit of analysis	Input and AI method	Sample/positive events	Validation design	Performance	Clinical interpretation and key limitations
Fu et al., 2025 (107)	Rectal cancer after curative-intent treatment	Local/distant recurrence-risk stratification; patient-level	Baseline MRI radiomics plus clinicopathological variables	n=600 (development n=358, internal test n=120 and geographically external test n=122), including 100 recurrence events overall (16.7%); cohort-specific event counts were not reported separately.	Three-centre development with internal and geographically external test cohorts.	All-Clin+Rad C-index 0.775 (95% CI, 0.723–0.827), 0.739 (95% CI, 0.654–0.823) and 0.822 (95% CI, 0.720–0.924) in the development, internal-test and external-test cohorts, respectively.	Provides externally tested stratification, but the surveillance action linked to the score remains undefined.
Xie et al., 2024 (108)	Early-onset rectal cancer	Composite recurrence risk; patient-level	MRI radiomics	n=264 patients with early-onset rectal cancer, including 60 patients with total recurrence (22.7%).	Three-centre development with internal and geographically external test cohorts.	EORC-ClinPathRadiom C-index 0.82 (95% CI, 0.75–0.88), 0.82 (95% CI, 0.74–0.91) and 0.81 (95% CI, 0.63–0.99) in the training, internal-test and external-test cohorts, respectively; corresponding recurrence HRs were 8.96, 6.81 and 7.46.	Population- and age-specific; should not be generalized without recalibration.
Sluckin et al., 2023 (109)	Rectal cancer with suspicious lateral nodes	Lateral locoregional recurrence; patient-level	Deep learning with explainability	n=196 from three Dutch tertiary centres; recurrence events NR.	Retrospective multicentre development with test-centre evaluation.	Combined imaging-clinical AUC 0.78 (95% CI, 0.60–0.96) for local recurrence and 0.80 (95% CI, 0.49–1.00) for lateral locoregional recurrence; For LR, sensitivity was 85.7% and specificity was 47.9%.	Selected high-risk cohort and low event counts limit transportability; explainability does not establish causality.
Liu et al., 2022 (110)	Rectal cancer	1-, 3- and 5-year recurrence-risk stratification; patient-level	DWI radiomics plus clinical variables	n=195: development cohort n=158, including 31 recurrence-or-metastasis events (19.6%), and geographically external cohort n=37, including 9 events (24.3%); total 40/195 (20.5%).	Two-centre development with geographically external centre validation.	Development-cohort AUCs were 0.887 (95% CI, 0.816–0.958), 0.813 (95% CI, 0.550–0.830) and 0.794 (95% CI, 0.655–0.932) at 1, 3 and 5 years, respectively; external-cohort AUCs were 0.819 (95% CI, 0.673–0.965), 0.795 (95% CI, 0.599–0.991) and 0.783 (95% CI, 0.580–0.987), respectively. C-index was 0.83 versus 0.68 for the clinical model.	Time-to-event calibration, competing risks and action thresholds require prospective evaluation.
Lee et al., 2020 (111)	Stage I-III CRC	5-year metachronous liver metastasis; patient-level	Preoperative liver CT CNN features plus clinical data	n=2,019, including 100 five-year metachronous liver-metastasis events (4.95%).	Single-centre repeated random hold-out evaluation: 70% training and 30% testing, with five-fold cross-validation for parameter tuning within each training set; the full split–tune–test procedure was repeated 100 times; no independent external validation.	Mean test-set AUC 0.747 ± 0.036 versus 0.709 ± 0.038 for clinical variables alone (mean ± SD across 100 repeated train/test iterations).	Moderate discrimination and no independent external validation; intended for surveillance research, not autonomous follow-up.
Taghavi et al., 2021 (112)	CRC after definitive treatment	Metachronous liver metastasis; patient-level	Staging CT radiomics plus clinical variables	n=91, including 24 metachronous liver-metastasis events within 24 months (26.4%).	Multicentre development/validation with a small validation cohort; centre independence was incompletely reported.	Validation AUC 0.86 (95% CI, 0.85–0.87) for both the radiomics and combined models, compared with 0.71 (95% CI, 0.69–0.72) for the clinical model.	Small sample; harmonisation, calibration, DCA and impact evidence absent.
Li et al., 2022 (113)	CRC after definitive treatment	Metachronous colorectal liver metastasis; patient-level	Primary-tumour CT radiomics-clinical fusion model	n=323: dataset 1 n=248, including 176 metachronous liver-metastasis events (71.0%), and geographically external dataset 2 n=75, including 23 events (30.7%); total 199/323 (61.6%).	Internal validation plus geographically external validation.	Fusion-model AUC 0.79 (95% CI, 0.68–0.87) internally and 0.72 (95% CI, 0.60–0.82) externally.	External performance was modest; no validated surveillance threshold. The marked difference in event prevalence between dataset 1 and the external dataset (71.0% vs 30.7%) indicates substantial spectrum shift and may affect calibration and transportability
Yao et al., 2026 (114)	CRC after surgery	Postoperative distant metastasis and 3-year DFS; patient-level multimodal model	CT deep-learning radiomics plus pathomics	n=521; events NR	Multicentre development/validation reported; validation AUC was unavailable in the abstract.	Training AUCs 0.914-0.931 for deep signatures; validation performance NR	Training metrics must not be treated as confirmatory; independent performance, calibration and DCA require reporting.
Fu et al., 2025 (115)	After radical resection of colorectal liver metastases	Early recurrence after metastasectomy; patient-level	CT radiomics tumour-quality/quantity model	n=282 from three centres: training n=138, internal validation n=60 and geographically external validation n=84; early-recurrence event count NR.	Three-centre retrospective study with training, internal-validation and external-validation cohorts.	RQQM AUC 0.88/0.88/0.84 and combined radiomics–clinical model AUC 0.89/0.89/0.86 in the training/internal/external cohorts; radiomics-only AUC 0.85/0.83/0.81.	Distinct post-hepatectomy population; not generalisable to stage I-III primary CRC.

AUC, area under the receiver operating characteristic curve; CEA, carcinoembryonic antigen; C-index, concordance index; CI, confidence interval; CRC, colorectal cancer; CRLM, colorectal liver metastases; DCA, decision-curve analysis; DFS, disease-free survival; EORC/LORC, early-/late-onset rectal cancer; LLN, lateral lymph node; LLR/LR, lateral locoregional/locoregional recurrence; RQQM, radiomics quality and quantity model. NR, not reported.

Because this was a structured narrative review rather than a systematic review, the search was not designed to produce an exhaustive record-level screening log or a formal count of included primary studies. Consequently, a PRISMA flow diagram and formal study-level risk-of-bias assessment were not undertaken.

C.D. and Y.L. independently extracted data using the prespecified evidence matrix, and discrepancies were resolved by discussion and consensus. The matrix comprised tumour site; clinical setting and intended decision; cohort size and positive-event count; input modality; endpoint and reference standard; unit of analysis and unit of data splitting; model type; internal, temporal, internal-external, geographically external or prospective validation; discrimination with confidence intervals where available; calibration; decision-curve analysis; reader or workflow evaluation; prospective clinical impact; model or code accessibility; and principal limitations. Pre-treatment, longitudinal and post-treatment inputs were recorded separately. Current-state inference, such as invasion-depth classification or synchronous-metastasis detection, was distinguished from future-event prediction, such as metachronous recurrence or survival.

### 2.4 Separation of study-level methodological support and task-level translational readiness

Study-level methodological support and task-level translational readiness were assessed separately. At study level, the review describes cohort representativeness, endpoint definition, reference standard, event prevalence, independence of data splitting, external validation, calibration, decision analysis and risk of data leakage. No formal study-level risk-of-bias score was assigned because the article was not designed as a systematic review. PROBAST+AI and TRIPOD+AI nevertheless informed the items extracted and the interpretation of model evidence (8, 9).

At task level, translational readiness was classified as higher, intermediate or early-stage. Higher readiness required consistent performance in representative multicentre data, independent external validation, clinically relevant thresholds, calibration or decision-analytic evaluation, and evidence of reader assistance, workflow use, prospective impact or implementation, together with practical model accessibility. Intermediate readiness indicated a replicated predictive signal with multicentre or external validation but incomplete calibration, action thresholds, accessibility, workflow testing or clinical-impact evidence. Early-stage evidence comprised predominantly retrospective single-centre studies, internal validation, small or selected cohorts, inconsistent external performance, unavailable models or absence of a defined clinical action. These categories describe maturity of a clinical task, not certainty that a model improves treatment outcomes. Positive-event counts and confidence-interval width were treated as measures of statistical precision. High point estimates derived from sparse validation cohorts or accompanied by wide confidence intervals were down-weighted and could not, by themselves, increase task-level readiness.

Prospective validation was defined as prospective application of a locked model to newly accrued patients without necessarily changing care. Prospective impact required evaluation of model-informed management against usual care or another prespecified comparator using patient, decision or workflow outcomes. Reader-assistance studies, publication of a nomogram or web calculator, and local implementation were not treated as equivalent to prospective impact. Two reviewers independently assigned the task-level categories using the prespecified matrix and resolved disagreements by consensus. DECIDE-AI informed interpretation of early clinical evaluation (10).

Evidence was synthesised qualitatively; no pooled effect estimates were calculated. Validation terminology was standardised as random-split internal validation, cross-validation, temporal validation, internal-external validation, geographically external validation or prospective validation. Patient-, lesion-, node-, image-, slide- and patch-level analyses were not pooled conceptually, because non-independent splitting at a level below the patient can inflate apparent performance. Common methodological limitations are consolidated in Section 7.2 rather than repeated after every subsection.

## 3 AI for pre-treatment staging and risk stratification

### 3.1 Site-specific applicability: colon cancer, rectal cancer, and mixed CRC cohorts

Colon and rectal cancers are commonly grouped as CRC, but they differ in anatomy, staging pathways, imaging, surgery, use of neoadjuvant treatment and the consequences of an incorrect prediction. Model performance must therefore be interpreted according to primary tumour site, assessment time point and intended decision (2, 3).

For colon cancer, contrast-enhanced CT is central to systemic staging and surgical planning, whereas pelvic MRI is not routinely used for local staging. In early T1 disease, post-endoscopic LNM risk may affect the decision for additional colectomy and lymph-node dissection. Rectal cancer follows a different pathway: pelvic MRI, mesorectal and lateral nodal assessment, nCRT or TNT, TME, local excision and watch-and-wait all depend on pre-treatment and post-treatment assessment. Consequently, rectal MRI nodal or response models cannot be extrapolated to colon CT pathways, and a high-risk result has different implications in colonic and rectal T1 lesions (11–17).

Accordingly, each study in this review is labelled as colon-specific, rectal-specific or broadly CRC-relevant. Models developed in mixed CRC cohorts are interpreted cautiously when colon and rectal results are not reported separately. Rectal MRI nodal or response models are not extrapolated to colon CT, and T1 CRC models used after endoscopic resection are interpreted according to the different consequences of additional colectomy, rectal excision, total mesorectal excision or organ-preservation pathways. Cross-study AUCs were not interpreted as a direct ranking of algorithmic superiority, because reported performance may vary according to the information content of the input modality, endpoint difficulty, cohort spectrum, event prevalence, unit of analysis and data splitting, reference standard, and validation design.

### 3.2 Optical invasion-depth assessment of already detected early lesions

This subsection concerns optical characterisation of an already detected colorectal lesion, not population-level polyp detection. The intended decision is whether endoscopic resection is likely to be oncologically appropriate or whether deep submucosal invasion should prompt surgical assessment. Because the reference standard, imaging mode and unit of analysis vary, performance for NICE/JNET classification, histological class, depth of invasion and curative-resection eligibility should not be treated as interchangeable endpoints. Published studies suggest that AI-assisted CAD systems may improve invasion-depth assessment, particularly when combined with image-enhanced endoscopy, magnifying endoscopy or endocytoscopy. Across published studies, reported diagnostic accuracies were generally between 80% and 90%, with some studies reporting values above 90%; Area under the curve (AUC) ranged from approximately 0.73 to 0.97 depending on imaging modality, lesion characteristics, outcome definitions and model architecture. A recent systematic review and meta-analysis further demonstrated that CAD algorithms achieved high pooled diagnostic accuracy and diagnostic odds ratios for predicting submucosal invasion, with performance approaching that of experienced endoscopists while providing greater benefit for less-experienced operators. These findings suggest that a potential clinical value of AI may lie in reducing expertise-related variability rather than replacing expert judgement (18).

Among representative studies, Tamura et al. evaluated the AI-assisted diagnostic system EndoBRAIN-Plus using a cohort of 66 pathologically confirmed lesions (event count: 18 T1b cancers, representing 27% prevalence). The AI-assisted modality achieved an overall diagnostic accuracy of 88.9% (95% CI: 86.3–91.2) and specificity of 93.1% (95% CI: 90.6–95.2) based on 660 image interpretations by 10 endoscopists, representing a significant improvement over white-light imaging alone (accuracy: 82.7% [95% CI: 81.2–86.9]). A key finding was the increase in high-confidence diagnoses from 40.2% with white-light imaging to 75.5% with AI support. For high-confidence cases specifically, AI assistance improved trainees’ diagnostic accuracy to 94.5% (95% CI: 90.8–97.0), comparable to experts’ performance of 96.2% (95% CI: 93.1–98.2). Notably, trainees’ sensitivity estimates showed wider confidence intervals (84.8% [95% CI: 71.1–93.7]) compared to experts (93.5% [95% CI: 84.3–98.2]), indicating more variable performance in detecting invasive cancer. Because the study did not evaluate multicentre transportability or model-guided treatment decisions, it cannot establish whether AI support translates into reduced unnecessary surgeries or improved clinical outcomes across institutions (19).

Endoscopic invasion-depth support was classified as intermediate in task-level readiness. The signal is supported by several image-based studies, reader comparisons and meta-analytic evidence, and its most plausible near-term value is reduction of expertise-related variability. Nevertheless, most studies used selected still images, and evidence that real-time model use reduces unnecessary surgery or missed deep invasion is lacking. Their near-term role is real-time reader assistance; prospective studies must determine whether they reduce missed deep invasion or unnecessary surgery. Histopathological confirmation remains required. Representative studies are summarised in **Table 1** (19–28).

### 3.3 Regional lymph-node assessment

Accurate prediction of regional lymph node metastasis (LNM) is central to several clinically distinct decision points in CRC. In early or T1 CRC after endoscopic resection, LNM risk influences whether additional radical surgery with lymph-node dissection is required. In rectal cancer, nodal status affects pre-treatment staging, the need for neoadjuvant chemoradiotherapy (nCRT) or total neoadjuvant therapy (TNT), surgical planning, and lateral lymph-node management. LNM prediction should therefore be interpreted according to tumour site, disease stage, imaging modality and intended clinical use rather than as a single generic CRC prediction task (3). Representative studies are summarised in **Table 2** (29–37).

#### 3.3.1 Pre-treatment rectal MRI nodal assessment

Among the reviewed approaches, MRI-based rectal-cancer models have a comparatively more mature evidence base. Xia et al. (29) developed the WISDOM model using a multicentre dataset of 1014 patients (training: n=589; internal test: n=146; external test cohorts: n=117 and n=162). For binary N staging (N0 versus N+), the model achieved an overall AUC of 0.81 (95% CI, 0.77–0.86); for ternary N staging (N0/N1/N2), it achieved an overall C-index of 0.765 (95% CI, 0.727–0.802). Model assistance significantly improved junior radiologists’ performance from an AUC of 0.69 (95% CI: 0.64–0.73) to 0.80 (95% CI: 0.76–0.84), and senior radiologists’ performance from 0.79 to 0.88 (95% CI: 0.85–0.91). However, the overall sensitivity remained relatively low at 70.2% (95% CI: 62.7%–77%), and the study was limited by its retrospective design and lack of node-by-node pathological matching. Wan et al. (30) developed a 3D ResNet101 model based on primary tumour T2-weighted MRI for predicting lymph node metastasis in stage T1–2 rectal cancer. In a single-centre test set of 86 patients (event count: 19 LNM positives, 22.1% prevalence), the model achieved an AUC of 0.79 (95% CI: 0.70–0.89). This performance was significantly superior to the pooled assessment of three radiologists (AUC 0.54 [95% CI: 0.48–0.60]; P< 0.001), driven by a marked improvement in sensitivity (100% vs. 26%). However, the model’s specificity was modest at 66%, and the absence of external multicentre validation limits the generalizability of these findings to broader clinical practice. Kasai et al. (31) developed a machine learning model for predicting lateral lymph node metastasis (LLNM) in lower rectal cancer using preoperative clinicopathological factors. In a single-centre temporal validation cohort of 107 sides (from 56 patients; event count: 10 LLNM-positive sides, 9.3% prevalence), the model achieved an AUC of 0.903 (95% CI: 0.832–0.974), significantly outperforming the conventional size-based method on MRI (AUC = 0.754; P = 0.022). Using a predefined cutoff, the model demonstrated 90.0% sensitivity and 79.4% specificity, correctly predicting four of five sides with LLNM measuring ≤4 mm, which are typically difficult to diagnose using size criteria alone. However, the limited number of positive events (n=10) in the validation set results in wide confidence intervals, and the absence of external multicentre validation limits the generalizability of the findings. Ao et al. (32) developed a two-centre retrospective deep-learning radiomics model using multiparametric MRI in 430 patients. In the external validation cohort (n=100), both the DLRS and combined nomogram achieved an AUC of 0.95, compared with 0.70 for the physician model. However, these unusually high estimates require caution because the model used manually selected primary-tumour images rather than direct lymph-node analysis, involved high-dimensional feature selection, and contained inconsistencies in performance reporting. The study is therefore considered supportive retrospective evidence requiring independent prospective validation, rather than decisive evidence of clinical readiness.

#### 3.3.2 Pre-treatment colon CT nodal assessment

By contrast, evidence for CT-based LNM prediction in colon cancer is substantially weaker. An informative negative example is the ResNet-50 model developed by Bedrikovetski et al. (34) to predict nodal status on preoperative CT in patients with colon cancer. In a large multicentre cohort of 1,201 patients, the model achieved an AUROC of only 0.619 (95% CI: 0.507–0.731) in the validation cohort (n=100). Performance deteriorated significantly in both the retrospective test cohort 1 (n=500; AUROC 0.542 [95% CI: 0.489–0.595]) and the prospective test cohort 2 (n=200; AUROC 0.486 [95% CI: 0.403–0.568]), approaching random classification. Despite high sensitivity (91.6%–96.6%), the model demonstrated extremely low specificity (5.2%–6.0%) in the test cohorts, indicating a failure to accurately identify node-negative patients and limiting its clinical utility for treatment stratification. This finding is clinically important because it demonstrates that AI does not automatically improve nodal staging and that models trained in one setting, tumour site, modality, or patient population may fail when transferred to external cohorts. It also reinforces the need to avoid extrapolating performance from rectal cancer MRI cohorts to colon cancer CT staging. For colon cancer, CT may provide useful information for distant metastasis and surgical planning, but current evidence does not support CT-based deep learning as a reliable stand-alone method for nodal staging (34, 38).

#### 3.3.3 Post-endoscopic-resection T1 CRC LNM risk

Whole-slide image (WSI)-based deep learning and clinicopathological machine-learning models provide a complementary pathway for LNM risk assessment, particularly in early or T1 CRC where the key clinical question is whether additional surgery is needed after endoscopic resection. Pathology-based models can extract features from routine H&E-stained slides, including glandular architecture, stromal response, immune infiltration, tumour budding-related morphology, and tumour microenvironment patterns. Zou et al. (37) developed a case-level multiple-instance learning framework using whole-slide histopathology images from 130 patients with T3/T4 colorectal cancer to predict lymph node metastasis (LNM). The CONCH v1.5 model achieved a mean AUC of 0.899 ± 0.033 with case-level training, compared with 0.814 ± 0.083 for slide-level training. Integration of pathological and clinical features yielded a mean AUC of 0.904 ± 0.047 for the best SVM model, compared with 0.584 ± 0.084 for the clinical-only model (mean ± SD across five-fold cross-validation). Because this cohort was not restricted to T1 disease or post-endoscopic-resection specimens, the study provides proof-of-concept for WSI-based LNM prediction rather than direct evidence for the additional-surgery decision in T1 CRC. T1-specific WSI-based and clinicopathological models have incorporated tumour differentiation, depth of invasion, lymphovascular invasion, tumour location, submucosal infiltration area, poorly differentiated clusters, serrated lesions, and serum markers. Shi et al. (36) developed a random forest model using clinical, endoscopic, and pathological variables to predict lymph node metastasis in 210 patients with T1 colorectal cancer (event count: 51 LNM positive, 24.3% prevalence). In the internal testing cohort (n=53), the model achieved an AUC of 0.872 (95% CI: 0.8158–0.8953), with 73.7% sensitivity and 90.0% specificity. The T1-specific models may be clinically relevant because they align more directly with post-endoscopic-resection decision-making than the broader CRC study by Zou et al. (37) or purely pre-treatment imaging models. However, most remain research-level tools, with small datasets, single-centre designs, cross-validation rather than independent external validation, and limited evidence that model-guided decisions reduce unnecessary surgery without increasing undertreatment.

These three nodal tasks have different inputs, reference standards and clinical consequences. Rectal-MRI nodal assessment was classified as intermediate because WISDOM provided multicentre external testing and reader-assistance evidence (29), with Wan et al. providing supportive evidence from a held-out single-centre test set (30). Kasai et al. (31) did not increase readiness because the temporal validation included only 10 LLNM-positive sides and had a wide confidence interval. Importantly, the rating remained unchanged after excluding Ao et al. (32). Clinical action thresholds and treatment-impact evidence nevertheless remain incomplete. Colon-CT nodal prediction and T1-CRC WSI or clinicopathological prediction were classified as early-stage: the former showed poor transportability, whereas the latter lacks evidence that model-guided use reduces unnecessary additional surgery without increasing undertreatment.

### 3.4 Synchronous distant metastasis: patient-level risk, lesion characterisation, and site-specific detection

This subsection is limited to metastatic disease present or suspected at initial staging. Models predicting metachronous metastasis after curative-intent treatment are discussed as recurrence models in Section 6.3, even when they use preoperative images. Three non-equivalent tasks are considered separately: patient-level estimation of occult synchronous metastasis, lesion-level characterisation of an already detected indeterminate lesion, and site-specific detection of metastatic deposits. Their AUCs should not be compared as if they represented one generic distant-metastasis prediction endpoint.

#### 3.4.1 Patient-level liver and multisite risk

The most frequently studied application is synchronous liver-metastasis risk stratification, particularly in rectal cancer. Liu et al. (39) developed a radiomics nomogram combining T2-weighted MRI features with serum biomarkers (CEA and CA19-9) to predict synchronous liver metastasis (SLM) in 127 patients with primary rectal cancer (event count: 32 SLM, 25.2% prevalence). In the training cohort (n=88), the nomogram achieved an AUC of 0.918 (95% CI: 0.824–1.000), which improved to 0.944 (95% CI: 0.895-0.993) in the validation cohort (n=39). This performance was significantly superior to the radiomics-only model in the validation set (AUC 0.866; P = 0.044). The model demonstrated high sensitivity (95.83%) and specificity (88.89%) in the validation set. However, the single-centre retrospective design and limited validation sample size restrict the generalizability of the findings. Mou et al. (40) developed a radiomics-clinical model combining endoscopic rectal ultrasound features with serum markers (CEA and CA19-9) to predict synchronous liver metastasis (SLM) in 239 patients with primary rectal cancer (event count: 34 SLM, 14.2% prevalence). In the training cohort (n=168), the combined model achieved an AUC of 0.920 (95% CI: 0.874–0.965), significantly outperforming the clinical-only model (AUC 0.811, P = .006). In the validation cohort (n=71), the model yielded an AUC of 0.855 (95% CI: 0.759–0.951), with a sensitivity of 100% and specificity of 67.7%. While the model demonstrated high sensitivity for screening, its performance in the validation set showed a drop in specificity and AUC compared to the training set, likely due to the small number of positive events (n=10 in validation set) and the single-centre retrospective design. Both studies were single-centre and are best interpreted as aids to staging review and multidisciplinary assessment rather than confirmation of metastatic disease.

Multimodal imaging may improve baseline risk estimation. Jiang et al. (41) developed a multiparametric MRI clinical-radiomics nomogram for synchronous distant metastasis (SDM) in 169 patients with rectal cancer (event count: 82 SDM, 48.5% prevalence; stratified into training: n=134 and test: n=35 cohorts). The nomogram, integrating radiomics features from T2-weighted and diffusion-weighted images with clinical risk factors (CEA, CA19-9, age, T stage), achieved AUCs of 0.93 (95% CI: 0.89–0.96) in the training cohort and 0.94 (95% CI: 0.79–0.97) in the test cohort. This performance was significantly superior to standalone radiomics or clinical models, demonstrating balanced sensitivity (85%-88%) and specificity (86%-89%). The model remains dependent on retrospective data and institution-specific acquisition and segmentation.

#### 3.4.2 Characterisation or detection of organ-specific lesions

Non-liver applications address narrower clinical problems. Hu et al. (42) developed a clinical-radiomics nomogram using CT texture features to distinguish metastatic from non-metastatic indeterminate pulmonary nodules (IPNs) in 194 patients with colorectal cancer (event count: 93 lung metastases, 47.9% prevalence; training cohort: n=136; validation cohort: n=58). The combined model integrated radiomics signature with clinical risk factors (N stage, chronicity) and achieved AUCs of 0.929 (95% CI: 0.885–0.974) in the training cohort and 0.922 (95% CI: 0.857–0.986) in the validation cohort, whereas Jin et al. (43) developed a random forest model based on GLCM features from DWI images to predict pelvic bone metastases in 614 colorectal cancer patients (event count: 53 bone metastases, 8.6% prevalence; training cohort: n=429; validation cohort: n=185). The model achieved AUCs of 0.926 (95% CI: 0.873–0.979) in the training set and 0.919 (95% CI: 0.868–0.970) in the internal validation set, significantly outperforming the generalized linear regression model (AUC 0.716). These selected cohorts do not establish general distant-metastasis screening, and low event numbers and site-specific reference standards limit interpretation.

Available models were classified as early-stage. Liver-risk nomograms may flag patients for closer review of staging examinations, whereas pulmonary-nodule classifiers address the narrower question of whether an existing nodule is metastatic, and pelvic-bone models address site-specific lesion detection. None demonstrates general multisite staging performance or an improvement in treatment sequencing, resectability assessment or survival. Standard imaging and multidisciplinary review therefore remain decisive (44). Representative studies are summarised in **Table 3**.

### 3.5 Why reported model performance differs across studies

Reported performance differences should not be interpreted as a direct ranking of algorithmic superiority because the studies varied substantially in clinical task, imaging modality, cohort characteristics and validation design. Algorithm architecture may contribute when models are compared within the same dataset; for example, Wan et al. found that a three-dimensional ResNet101 achieved the best test AUC, suggesting a potential benefit from volumetric information (30). However, the information contained in the input may be more influential: narrow-band imaging substantially outperformed white-light imaging within the same endoscopic study (20), while multiparametric MRI and clinical-radiomics models may benefit from combining anatomical, diffusion and clinical information (29, 32, 39–41). Dataset selection and study design also affect apparent performance. The AUC of 0.994 reported from selected MRI screenshots in a small single-centre cohort (35) contrasts with the more moderate but multicentre externally tested WISDOM AUC of 0.81 (29) and the near-chance test performance of a large colon-CT ResNet-50 model (34). Endpoint difficulty is another important factor, as Luo et al. reported an AUROC of 0.970 for a broad invasion category but only 0.729 for the more difficult superficial-versus-deep invasion task (22). Thus, current differences are more likely attributable to the combined effects of modality, endpoint definition, cohort spectrum, unit of analysis and validation rigor than to architecture alone, and higher internal AUCs should not be considered evidence of superior clinical value without independent external validation (20, 22, 29–43).

## 4 Molecular-biomarker pre-screening and treatment-response stratification

Molecular-biomarker inference and treatment-response prediction are related but distinct tasks. MSI-H/dMMR status has established clinical implications in CRC and is conventionally determined by IHC, PCR-based testing or next-generation sequencing (NGS). TMB is a sequencing-derived genomic quantity whose independent predictive value, particularly in microsatellite stable/proficient mismatch repair CRC, remains less certain. In this section, image- or morphology-derived outputs are evaluated as pre-screening or testing-prioritisation signals, whereas IHC-based systems are evaluated as workflow-automation tools (54, 55). Biomarker status still requires confirmation by IHC, PCR or NGS, and treatment-specific models require prospective decision-impact evaluation.

### 4.1 Image-derived MSI/MMR pre-screening

Among molecular biomarker applications in colorectal cancer, AI-based prediction of microsatellite instability (MSI) and mismatch-repair deficiency (dMMR) is one of the more extensively studied areas with potential clinical relevance. MSI/MMR status has implications for Lynch syndrome screening, prognosis, adjuvant-treatment considerations and immunotherapy selection. However, AI-based MSI/MMR prediction should be clearly distinguished from conventional molecular testing. IHC directly evaluates MMR protein expression, PCR-based assays detect MSI at selected loci, and NGS assesses MSI or related genomic alterations at the molecular level. By contrast, AI models infer the probability of MSI-H/dMMR from indirect phenotypic correlates, including radiological tumour heterogeneity, metabolic activity, histological architecture, immune infiltration, stromal response and tumour-microenvironment patterns. Accordingly, AI-positive cases require confirmatory IHC, PCR or NGS, while the safety of AI-negative cases requires prospective evaluation (54).

#### 4.1.1 Radiological MSI/MMR risk estimation

Radiomics-based MSI/MMR prediction has been explored using CT, MRI and PET/CT. Earlier preliminary CT radiomics work also reported potential MSI-predictive features in stage II CRC (46). CT radiomics has the advantage of broad clinical accessibility because contrast-enhanced CT is routinely used in CRC staging. Cao et al. (48) developed a multicentre triphasic enhanced CT radiomics model in 502 patients with CRC, including 441 patients in the training cohort and 61 in the external validation cohort. The delayed-phase CT radiomics model outperformed arterial- and venous-phase models, and the combined clinical-radiomics nomogram incorporating age, tumour location and CEA achieved an AUC, accuracy and sensitivity of 0.898 (95% CI: 0.860–0.937), 0.837 (95% CI: 0.799–0.870) and 0.821 (95% CI: 0.672–0.896) in the training cohort, respectively, and 0.964 (95% CI: 0.919–1.000), 0.918 (95% CI: 0.819–0.973) and 1.000 (95% CI: 0.667–1.000) in the external validation cohort, respectively. Bian et al. (49) also developed a multiphasic enhanced CT radiomics nomogram in 347 patients with CRC (event count: 71 MSI, 20.5% prevalence; training cohort: n=243; testing cohort: n=104). The final three-phase combined model (AP+VP+DP) outperformed single-phase models, achieving AUCs of 0.827 (95% CI: 0.763–0.891) in the training set and 0.714 (95% CI: 0.604–0.825) in the testing set. The nomogram, integrating the radiomics score with independent clinical predictors (PLT, SII, tumour location, enhancement pattern, and ACR), yielded AUCs of 0.894 (95% CI: 0.848–0.939) and 0.839 (95% CI: 0.738–0.940) in the training and testing sets, respectively. These studies suggest that CT radiomics may provide a non-invasive MSI/MMR screening signal, but its clinical use remains limited by retrospective design, heterogeneous CT protocols, relatively small external test sets and the need for standardised feature extraction and calibration.

MRI-based MSI/MMR prediction has been investigated mainly in rectal cancer, where pelvic MRI is already central to local staging. MRI may capture local tumour architecture, diffusion restriction and intratumoural heterogeneity more directly than CT. Jing et al. (50) developed multiparametric MRI radiomics models in rectal cancer using T2-weighted imaging, diffusion-weighted imaging, contrast-enhanced T1-weighted imaging and multi-sequence combinations, with cohorts from two hospitals. The multi-sequence model outperformed single-sequence models, suggesting that anatomical, diffusion and contrast-enhanced information may be complementary. Li et al. (51) conducted a retrospective multicentre study of 199 patients with rectal cancer (event count: 68 MSI-H, 34.2% prevalence; training cohort: n=100 from Hospital I; external validation cohort: n=99 from Hospitals II & III). While clinical features (age, gender, CEA, CA19-9) showed no significant differences between MSI groups, the CE-T1WI single-sequence model achieved AUCs of 0.78 (95% CI: 0.685–0.869) in the training set and 0.77 (95% CI: 0.667–0.875) in the external validation set. Similarly, the multi-sequence combined model achieved AUCs of 0.78 (95% CI: 0.680–0.872) in both the training and external validation sets. In contrast, models based on unenhanced sequences (T1WI, T2WI) showed a significant drop in AUC during external validation (e.g., T2WI: 0.71 to 0.66), suggesting that contrast-enhanced imaging provides superior generalizability. More recently, Cai et al. (52) shifted from whole-tumour radiomics to subregion-based MRI radiomics in a multicentre cohort of 475 patients with rectal cancer (event count: 53 MSI, 11.16% prevalence; training cohort: n=382 from Center A; external test cohort: n=93 from Center B). The subregion model achieved AUCs of 0.86 (95% CI: 0.79–0.93) and 0.83 (95% CI: 0.74–0.92) in the training and external testing cohorts, respectively, significantly outperforming the conventional whole-tumour radiomics model (AUC 0.72 and 0.73, P<0.05). When combined with clinicoradiological variables (tumour location, N stage), the optimal model yielded AUCs of 0.87 and 0.85 in training and external test cohorts, respectively, and successfully stratified patients by 3-year disease-free survival (predicted MSI vs. MSS: 86% vs. 35%, P = 0.032). After integration with clinical and radiological variables, the combined subregion-clinical-radiological model achieved AUCs of 0.87 and 0.85. These findings suggest that MRI radiomics may support rectal cancer-specific MSI/MMR risk stratification, but current performance remains insufficient for replacing standard molecular testing, and the field still requires prospective validation, sequence standardisation and external testing across scanners and institutions.

PET/CT radiomics provides a complementary route because it combines anatomical and metabolic information. Kim et al. (45) evaluated ¹^8^F-FDG PET/CT radiomics in 233 patients with CRC (event count: 25 MSI-high, 10.7% prevalence; training set: n=139; test set: n=94). The radiomics score (rad_score) constructed from two features (MinorAxisLength, JointEntropy) achieved AUROCs of 0.815 (95% CI: 0.713–0.917) and 0.867 (95% CI: 0.757–0.977) in the training and test sets, respectively. This model significantly outperformed conventional metabolic tumour volume (MTV) in the test set (AUC 0.794, P = 0.015) and proved to be an independent predictor of MSI status (OR: 1.012, P = 0.004). This suggests that PET/CT radiomics may capture MSI-related metabolic heterogeneity more sensitively than standard metabolic-volume metrics. However, PET/CT is not universally obtained for all patients with CRC, and available evidence remains limited in sample size and validation breadth. Its most plausible role is therefore adjunctive biomarker risk stratification in selected imaging contexts, rather than routine MSI/MMR screening for all patients.

Radiological models were classified as early-stage. They may provide an opportunistic risk signal from examinations obtained for staging, but scanner dependence, selected cohorts and limited external calibration currently restrict these models to opportunistic risk estimation.

#### 4.1.2 H&E whole-slide-image pre-screening

Compared with radiomics, H&E whole-slide-image-based deep learning is closer to the biological substrate of MSI/MMR status because it directly analyses tumour morphology and the immune-tumour microenvironment on routine histopathology slides. WSI models can extract features related to glandular architecture, lymphocytic infiltration, stromal response, mucinous components, necrosis and spatial organisation of the tumour microenvironment. Schirris et al. (53) proposed DeepSMILE, a contrastive self-supervised and weakly supervised framework for MSI prediction directly from H&E WSIs (n=360 patients). In five-fold cross-validation on TCGA-CRC, ImageNet tile supervision achieved a mean AUROC of 0.77 ± 0.06, SimCLR tile supervision achieved 0.87 ± 0.01, and the slide-level SimCLR–VarMIL DeepSMILE model achieved 0.86 ± 0.02 (mean ± SD). These findings support the feasibility of morphology-based MSI prediction, but they remain primarily model-development evidence and require evaluation in clinically representative pathology workflows.

MSIntuit represents an independently validated example of a clinically oriented AI pre-screening tool for MSI detection from CRC histology slides. Saillard et al. (54) validated MSIntuit after training on TCGA samples (n=859 WSIs) and testing it in an independent blinded validation cohort of 600 consecutive patients with CRC. The model achieved high sensitivity of 0.96–0.98, but specificity was modest at 0.46–0.47, resulting in an AUC of 0.88 (95% CI: 0.85–0.91) across both scanners. This performance profile is important clinically: high sensitivity supports a possible pre-screening or triage role, but limited specificity means that many AI-positive cases would still require confirmatory testing. The principal near-term value of MSIntuit is prioritising cases for confirmatory MSI/MMR testing.

H&E WSI-based MSI/MMR pre-screening was classified as intermediate in task-level readiness. MSIntuit provides independent blinded validation and a clinically plausible high-sensitivity triage profile, but its modest specificity means that AI-positive cases still require confirmatory testing and AI-negative cases require safety evaluation across populations, scanners and staining workflows. H&E morphology-based models infer a molecular phenotype; they do not directly measure MMR protein expression or microsatellite instability.

### 4.2 IHC-based MMR protein-expression workflow automation

AIMMeR represents a different application from H&E morphology-based MSI prediction. It analyses MMR-protein IHC at single-cell level to automate assessment of protein-expression patterns in routine pathology samples. In two independent CRC clinical-trial cohorts (SCOT: n=685; QUASAR2: n=381), AIMMeR achieved an AUROC of 0.98 (95% CI: 0.97–0.99) relative to pathologist assessment, with specificity of 75–77% at 98% sensitivity and a positive predictive value of ≥98% for the commonest pattern of somatic MMR deficiency (combined MLH1-PMS2 loss). Inter-rater reliability was substantial (Cohen’s κ: 0.79–0.82). Concordance with MSI-PCR was lower (AUROC 0.86, 95% CI: 0.82–0.90), which is biologically plausible because IHC protein-expression status and PCR-defined microsatellite instability are related but non-identical reference standards. AIMMeR should therefore be described as IHC workflow automation and decision support, not as H&E WSI inference of MSI. It was classified as intermediate in task-level readiness because independent cohort evaluation exists, but prospective workflow, safety and impact evidence remain limited due to reliance on TMA cores rather than whole slides and absence of germline/somatic sequencing for aetiology confirmation (55).

### 4.3 TMB prediction from histology and multimodal data

TMB is conventionally quantified by sequencing and is affected by platform, panel size, tumour purity, tissue quality, bioinformatic pipeline and the threshold used to define TMB-high status. Morphology- or clinical-data-based prediction is therefore indirect risk estimation rather than molecular diagnosis. Its proposed near-term use is sequencing prioritisation; no validated action threshold currently links an image-derived TMB probability to immunotherapy eligibility (47).

Shimada et al. (56) developed a CNN-based model to predict TMB-high (TMB-H) CRC directly from H&E slides in a combined cohort of Japanese CRC (JP-CRC, n=201) and TCGA-CRC (n=77) patients. The model achieved a mean AUC of 0.934 (range: 0.835–0.981), with the best-performing model reaching an AUC of 0.981 (sensitivity: 1.000; specificity: 0.923). Tumour-infiltrating lymphocytes (TILs) were identified as an independent predictor for TMB-H (OR: 29.26, P = 0.003), yielding an AUC of 0.910 for manual prediction. The study was limited by a small sample size (n=17 TMB-H cases) and lack of external validation, necessitating further prospective studies. Huang et al. (57) combined histopathological images and clinical information from a TCGA CRC cohort (n=509) using a multi-modal compact bilinear pooling (MCB) fusion framework. With TMB-high defined as the top 14.3% of the cohort (73/509; threshold ≥20 mutations/Mb), the multimodal ResNet18-based model achieved an AUC of 0.817, accuracy of 0.872 and recall of 0.676 after five-fold cross-validation, compared with an AUC of 0.804 for the image-only model. The primary report did not provide confidence intervals for these estimates. Wang et al. (58) applied an ensemble transformer-based MIL framework (ETMIL-SSLViT) to TCGA cohorts (EC: n=918 WSIs/529 patients; CRC: n=1495 WSIs/594 patients). Integrating a self-supervised ViT encoder, it achieved superior performance in EC subtype classification (AUROC: 0.92; Accuracy: 91%; MeanSS: 90%) and aggressive EC TMB prediction (AUROC: 0.83; Accuracy: 77%; MeanSS: 77%), outperforming seven SOTA methods. For CRC, it achieved an AUROC of 0.87 for subtype classification and 0.90 for non-mucinous TMB prediction. The model is computationally efficient, requiring <3 hours for training and ~27 seconds per WSI for inference on a GTX 1080 Ti GPU. These studies support a learnable signal but do not establish interchangeability with sequencing. Because MSI-H/dMMR CRC is commonly TMB-high and shares recognisable H&E features, including lymphocyte-rich and mucinous morphology, models developed in unstratified cohorts may partly learn MSI-associated morphology rather than an independent TMB signal. Shimada et al. (56) reported a limited supplementary analysis separating MSI-H/TMB-H and MSS/TMB-H test cases across 10 random splits; however, this small internal analysis did not establish pMMR-specific or externally reproducible performance. Huang et al. (57) and Wang et al. (58) did not report TMB-prediction performance stratified by MSI/MMR status. Thus, current evidence does not establish MSI/MMR-independent TMB prediction. Future studies should prespecify MSS/pMMR subgroup analyses, report subgroup event counts and confidence intervals, and evaluate biologically distinct MSS/pMMR TMB-high tumours. External reproducibility, threshold definition, calibration and clinical action also remain uncertain.

AI-based TMB prediction was classified as early-stage. Available studies are retrospective, use heterogeneous TMB-high thresholds and provide limited external validation or clinical-impact evidence. External reproducibility, calibrated sequencing-prioritisation thresholds and clinical-impact evidence remain absent.

### 4.4 Early evidence for AI-supported neoadjuvant immunotherapy-response prediction

Direct evidence remains limited to a small number of highly selected, treatment-specific cohorts. This task is distinct from MSI/MMR or TMB prediction because actual response depends on molecular status, immune-cell infiltration, stromal architecture, tumour burden, treatment regimen and clinicopathological context. The evidence was therefore classified as early-stage (59).

Zhang et al. (60) developed an immunophenotype-guided radiomics model for response to neoadjuvant anti-PD-1 therapy in stage III-IV dMMR/MSI-H CRC. RNA-sequencing and IHC were used to define immune phenotypes that were linked to imaging features, providing a biologically informed proof of concept. However, the selected molecular subgroup and regimen-specific setting limit generalisability, and the model has not been shown prospectively to improve patient selection, treatment duration or surgical planning.

No prospective evidence supports using these models to change immunotherapy eligibility or surgical planning. The field requires treatment-specific prospective cohorts and standardised radiological and pathological response endpoints before clinical utility can be assessed. Taken together, current evidence supports AI primarily as a complementary tool rather than a reliable alternative to established molecular diagnostics. Imaging- and H&E-based MSI/MMR models infer phenotypic correlates and may support pre-screening or testing prioritisation, but positive results require confirmation by IHC, PCR or NGS, while the safety of AI-negative triage requires prospective evaluation (48, 54). AIMMeR is better regarded as automation of MMR-IHC interpretation rather than a replacement for molecular testing (55). TMB and immunotherapy-response models remain exploratory because biomarker thresholds vary, external validation and calibration are limited, MSI/MMR-associated morphology may confound TMB prediction, and no prospective evidence demonstrates that model-guided use improves treatment selection or patient outcomes (56–58, 60). Accordingly, the most plausible current applications are testing prioritisation, pathology workflow support and biomarker-stratified research; replacement of established assays or independent treatment selection is not currently justified.

## 5 AI for treatment-response prediction in rectal cancer

nCRT and TNT are integral to the management of locally advanced rectal cancer. Artificial-intelligence-based models have been developed for two clinically distinct purposes: prediction of pathological response from information available before treatment and assessment of residual disease after treatment. These tasks should not be conflated. pCR is confirmed in a resection specimen, whereas cCR is determined without surgery and is the relevant state for considering watch-and-wait or other organ-preservation strategies.

AI may provide additional quantitative information from MRI, CT, PET/CT, pretreatment biopsy slides, endoscopy and longitudinal biomarkers. However, prediction of response is not itself a treatment intervention. No reviewed study demonstrated that model-guided response assessment improved treatment selection, organ-preservation safety or patient outcomes.

### 5.1 Pre-treatment prediction of pathological response

Pre-treatment models use only information available before nCRT or TNT. Their intended role is to estimate the probability of pCR or favourable pathological response before treatment begins. Their current use is pretreatment risk stratification; thresholds for treatment escalation, de-escalation, omission of surgery or watch-and-wait have not been clinically evaluated.

Baseline MRI radiomics is the most frequently studied approach. T2-weighted imaging, diffusion-weighted imaging and dynamic contrast-enhanced MRI may quantify tumour morphology, cellularity, perfusion and spatial heterogeneity. In an intercontinental external validation (n=59), the baseline MRI model evaluated by Boldrini et al. achieved an AUC of 0.83 (95% CI: 0.71–0.96). At the previously specified threshold, sensitivity was 0.70 (95% CI: 0.35–0.93), specificity was 0.64 (95% CI: 0.49–0.77), positive predictive value was 0.28 (95% CI: 0.18–0.40), and negative predictive value was 0.91 (95% CI: 0.80–0.97). Because pCR was the positive outcome, the high negative predictive value mainly indicates an ability to identify patients unlikely to achieve pCR; the low positive predictive value limits its use for confirming pCR or selecting treatment de-escalation (61).

The radiopathomics integrated prediction system developed by Feng et al. combined pretreatment MRI radiomics with nuclear and tumour-microenvironment features extracted from pretreatment H&E-stained biopsy slides. RAPIDS was developed in 303 patients and evaluated in two retrospective external cohorts of 480 and 150 patients and a prospective observational cohort of 100 patients. The corresponding AUCs were 0.868 (95% CI, 0.825–0.912), 0.860 (0.828–0.892), 0.872 (0.810–0.934), and 0.812 (0.717–0.907), respectively. In the prospective cohort, sensitivity was 0.888 (95% CI, 0.728–0.999), specificity was 0.740 (0.593–0.886), positive predictive value was 0.512 (0.313–0.710), and negative predictive value was 0.929 (0.862–0.995). These multicentre external and prospective observational results provide comparatively strong validation of predictive discrimination. However, adherence to reporting guidelines does not itself establish low risk of bias, model robustness or clinical effectiveness. RAPIDS was not used to allocate treatment and therefore was not evaluated in a prospective clinical-impact study (62).

More recent baseline MRI models have incorporated subregional radiomics, deep-learning features and automated feature extraction. Wu et al. (63) developed the SRADL (Sub-regional Radiomics And Deep Learning) model in a training cohort of 401 patients (pCR rate: 31.7%) and evaluated it in two external cohorts of 209 and 158 patients, with AUCs of 0.925 (95% CI: 0.894–0.948), 0.915 (95% CI: 0.869–0.949) and 0.902 (95% CI: 0.846–0.945), respectively. The model integrated sub-regional radiomics (based on K-means clustering) and deep learning features (from 3D ResNet50), demonstrating superior performance compared to conventional radiomics (AUC 0.777-0.794) or deep learning alone (AUC 0.786-0.854). In the training cohort, the SRADL model achieved a sensitivity of 0.945, specificity of 0.788, and NPV of 0.969. Limitations included its retrospective design and lack of prospective validation, which warrants further investigation. Yardimci et al. (64) also reported internally validated discrimination using pretreatment sagittal T2-weighted MRI. These studies support the feasibility of baseline MRI risk stratification, although differences in MRI acquisition, segmentation, treatment regimen and endpoint definition continue to limit direct comparison.

CT and PET/CT may provide complementary baseline information. Pretreatment contrast-enhanced CT radiomics and CT-based fractal analysis have been evaluated for response prediction, while baseline ^18F-FDG PET/CT radiomics may add information on tumour metabolism. These modalities are attractive when CT is already available for staging or MRI quality is inadequate, but the supporting studies are generally smaller and less frequently externally validated than the MRI literature. Their smaller cohorts, limited external validation and protocol dependence currently restrict their transportability relative to pelvic MRI (65–67).

Pretreatment pathomics provides another non-radiological route. Zhang et al. (68) analysed pre-nCRT H&E-stained biopsy specimens from 294 patients at two institutions. The XGBoost model achieved an AUC of 1.000 in the development cohort. The abstract reported an external AUC of 0.950, whereas the detailed Results section reported an AUC of 0.908 (95% CI, 0.815–1.000) in the 50-patient external cohort, which contained only eight pCR events. The discrepancy between the abstract and Results, the small number of external events and the perfect development performance indicate a substantial risk of overfitting and require cautious interpretation.

Meta-analyses indicate favourable pooled discrimination but also substantial methodological heterogeneity. One MRI-focused analysis of 21 studies and 1873 patients reported pooled sensitivity of 0.82, specificity of 0.86 and a summary AUC of 0.91 (69). A later multimodality analysis of 34 studies and 9933 patients reported pooled sensitivity of 82%, specificity of 84% and an AUC of 0.90 (70). These pooled estimates should not be interpreted as evidence that one transportable clinical model has achieved these values. Many contributing studies were retrospective, single-centre and internally validated, and differed in treatment regimen, prevalence of pCR, imaging acquisition, segmentation and reference-standard assessment.

At the task level, pre-treatment pCR prediction was classified as intermediate in translational readiness because several models have undergone multicentre, external or prospective validation. The rating was based principally on the larger external and prospective observational studies (61, 62). The external estimate reported by Zhang et al. (68), which was based on only 8 pCR events and had a wide confidence interval, was down-weighted and did not determine the Intermediate rating. This classification reflects predictive validation rather than demonstrated treatment benefit. Transportability across conventional nCRT, induction-TNT, consolidation-TNT and immunotherapy-containing regimens should not be assumed, and pre-treatment pCR probabilities should not be used as evidence of cCR. Representative baseline models are summarised in **Table 4**.

### 5.2 Post-treatment and longitudinal response assessment

Models that incorporate restaging MRI, paired baseline and post-treatment imaging, post-treatment tumour measurements or dynamic biomarkers address a different clinical question from pure pre-treatment prediction. They estimate treatment response after exposure to nCRT or TNT and should be classified as post-treatment or longitudinal models even when part of their input was acquired at baseline.

#### 5.2.1 Post-treatment pCR estimation and reader support

Post-treatment MRI can be difficult to interpret because fibrosis, oedema, mucinous change and small residual tumour foci may produce overlapping appearances. Horvat et al. evaluated post-treatment T2-weighted MRI radiomics in 114 patients (pCR rate: 18%). The random forest model achieved an AUC of 0.93 (95% CI: 0.87, 0.96), sensitivity of 100% (95% CI: 0.84, 1.0), specificity of 91% (95% CI: 0.84, 0.96), positive predictive value of 72% (95% CI: 0.53, 0.87), and negative predictive value of 100% (95% CI: 0.96, 1.0). A subsequent external validation study used an independent cohort of 50 patients and combined radiomics with qualitative radiologist assessment; the combined model increased inter-reader agreement (κ=0.82 versus 0.25) and slightly improved the junior radiologist’s specificity, positive predictive value and negative predictive value. These studies support AI as reader assistance for identifying possible residual disease, but their pathology-based reference standard does not establish the safety of selecting cCR for non-operative management (71, 125).

RAPIDS-II provides stronger prospective reader-assistance evidence. The model combined radiomics from post-neoadjuvant-therapy MRI with clinicopathological variables. It was developed and tested retrospectively in 823 patients, divided into training and test cohorts of 575 and 248 patients. The test-set AUC was 0.813 (95% CI, 0.736-0.874). In a multicentre prospective validation cohort of 207 patients, the AUC was 0.795 (95% CI, 0.723-0.859). Model assistance improved the overall AUC and sensitivity of radiologists’ visual assessment, particularly for junior radiologists. This study therefore supports prospective model validation and human–model interaction, but it did not test whether model-guided management improved organ preservation, salvageability or patient outcomes (72).

Several studies previously described as pre-treatment models used mixed-timepoint inputs and are more appropriately considered here. Cheng et al. evaluated multiparametric MRI-based radiomics (T1W, T2W, and T2FS) in 193 patients. Radiomics features were extracted from baseline MRI, but the final pCR nomogram also included post-treatment tumour length and thickness; it is therefore not a purely pretreatment model. The reported validation AUC of 0.912 (95% CI: 0.843-0.982) for pCR applies to this mixed-timepoint nomogram (73).

Xia et al. developed Rectal-RadioSAM, an automated MedSAM–radiomics/XGBoost pipeline using paired baseline and post-nCRT T2-weighted and logarithmic diffusion-weighted MRI in 378 patients. In the reconciled single-centre held-out test cohort of 114 patients, including 22 pCR and 92 non-pCR cases, the combined pre- and post-treatment pipeline achieved a mean AUC of 0.83 ± 0.04 (SD) and a mean accuracy of 0.79 ± 0.04 (SD). The pipeline may reduce manual segmentation burden, although the low post-treatment DWI Dice coefficient and absence of geographically external validation limit its current generalisability (74).

Dynamic tumour-marker models are also longitudinal. Chen et al. constructed a dynamic tumour marker score (DTMS) based on changes in CEA and CA19–9 levels before and after nCRT in 1316 patients. In the 624-patient training subset, tenfold cross-validation yielded a mean AUC of 0.86 and an AUPRC of 0.732. The XGB model achieved AUCs of 0.80 and 0.82 and AUPRCs of 0.519 and 0.593 in the two geographically external validation cohorts, respectively. The primary report did not provide confidence intervals for these AUC estimates. These results suggest that response assessment may benefit from temporal biomarker information, but such a model cannot inform the initial treatment choice because its key input becomes available only after treatment has begun or been completed (75).

Post-treatment pCR assessment and reader support were classified as intermediate in task-level translational readiness because externally and prospectively validated human–model interaction evidence is available. The available human–model evidence concerns pCR reader support and does not establish cCR or organ-preservation safety.

#### 5.2.2 cCR assessment and organ-preservation safety

The clinically relevant endpoint for watch-and-wait is cCR or a carefully monitored near-cCR, not model-predicted pCR. Current assessment requires integration of digital rectal examination, endoscopy, pelvic MRI including diffusion-weighted imaging, tumour markers and serial reassessment. Combined clinical, endoscopic and MRI assessment provides the foundation for selecting patients for organ-preservation pathways, and recent consensus work has sought to standardise the definition of near-cCR (76, 77).

Endoscopic AI has been investigated as a potential adjunct. Haak et al. (78) evaluated deep learning on post-neoadjuvant therapy endoscopic images in 226 patients (48% CRs). The combined model using endoscopic images and clinical variables achieved AUCs ranging from 0.76 to 0.83. The EfficientNet-B2 model demonstrated the highest performance with an AUC of 0.83 (95% CI: 0.80–0.86), accuracy of 0.75 (95% CI: 0.72–0.79), sensitivity of 0.77 (95% CI: 0.73–0.80), and specificity of 0.75 (95% CI: 0.72–0.79). Williams et al. (79) developed a convolutional neural network (CNN) using 2,717 endoscopic images from 288 patients with locally advanced rectal cancer treated with total neoadjuvant therapy (TNT). The ResNet-50 model achieved AUROCs of 0.98 in the main test set and 0.92 in the local-regrowth test set; the primary report did not provide confidence intervals for these AUROC estimates. Corresponding accuracies were 0.91 (95% CI, 0.89–0.93) and 0.78 (95% CI, 0.70–0.84), respectively. Although the model performed similarly to surgeons for identifying non-complete response after TNT, all clinician groups outperformed it for local-regrowth images. This distinction is important because failure to detect local regrowth may delay salvage treatment.

The clinical evidence supporting organ preservation derives from watch-and-wait cohorts and TNT trials rather than from AI studies. The International Watch & Wait Database demonstrated that carefully selected patients with cCR could avoid immediate radical resection, while also showing the need for intensive surveillance for local regrowth and distant metastasis. The OPRA trial established a response-adapted pathway in which cCR and near-cCR were associated with a greater probability of organ preservation, whereas incomplete response generally prompted total mesorectal excision. These studies define the clinical pathway in which AI might be evaluated, but they do not validate AI-based selection (80, 81).

AI-assisted cCR and organ-preservation selection remains early-stage in translational readiness. Future studies should use standardised cCR and near-cCR definitions and evaluate missed residual disease, local regrowth, time to salvage surgery, R0 salvage rate, distant metastasis, organ-preservation survival and patient-reported outcomes. Before model outputs enter watch-and-wait selection, prospective studies must quantify missed residual disease, local regrowth and salvage outcomes (76–81). Post-treatment and longitudinal models are summarised in **Table 5**.

Beyond discrimination, the clinical utility of treatment-response models depends on calibration, threshold-specific predictive values, decision-curve net benefit, incremental value over conventional assessment and the consequences of prediction errors. The reviewed studies illustrate why AUC alone may be misleading: an external AUC of 0.83 was accompanied by a positive predictive value of only 0.28, while prospective validation of RAPIDS yielded an AUC of 0.812, a positive predictive value of 0.512 and a negative predictive value of 0.929 (61, 62). Some models improved inter-reader agreement or radiologist sensitivity (72, 125), and decision-curve analysis suggested potential net benefit in selected studies (63, 64); however, these remain surrogate indicators of utility. Most models used pathological complete response after surgery as the reference standard, which does not establish the safety of identifying clinical complete response or selecting patients for watch-and-wait. No reviewed study evaluated whether a predefined model-guided pathway reduced missed residual disease, increased safe organ preservation, improved salvageability or improved patient-reported or survival outcomes. The currently supported role is therefore reader assistance and response-risk stratification, while prospective clinical-impact studies with prespecified action thresholds are still required.

## 6 Perioperative risk and postoperative prognosis

### 6.1 Perioperative risk prediction

Perioperative models were classified according to their intended endpoint. Mortality-risk models and implemented perioperative pathways are discussed separately from complication-specific models because their validation requirements, potential actions and consequences differ substantially.

#### 6.1.1 Short-term mortality prediction and implemented pathways

Large registry studies provide the most substantial evidence for short-term mortality prediction. A Danish national study of 65,612 patients developed models for 30-, 90- and 180-day mortality, with AUCs of 0.871 (95% CI: 0.86–0.882), 0.874 (95% CI: 0.864–0.882), and 0.876 (95% CI: 0.867–0.883) (82). A Dutch cohort of 62,501 patients similarly evaluated 30-day mortality using 103 preoperative variables. These studies demonstrate that registry data can support mortality-risk estimation, but retrospective or registry validation alone does not show that presenting the prediction to clinicians changes care or improves outcomes (83).

Rosen et al. (84) moved beyond model development by embedding a 1-year mortality model in personalised perioperative pathways within a Danish hospital. In a non-randomised before-and-after implementation study, patients managed through the pathway had a lower incidence of a comprehensive complication index greater than 20 than patients receiving standard care, and short-term economic modelling suggested potential cost-effectiveness. This is an important workflow-implementation example. Nevertheless, the intervention combined AI-based risk prediction with a multicomponent care pathway; therefore, the observed differences cannot be attributed to the prediction model alone. Secular changes, patient mix and other components of the pathway may have contributed.

Mortality models may inform counselling, prehabilitation, anaesthetic planning and monitoring; however, no validated threshold currently supports using predicted mortality to deny surgery. Because predicted mortality is strongly influenced by age, frailty, comorbidity and healthcare access, subgroup calibration and fairness assessment are essential. Short-term mortality decision support was classified as intermediate in translational readiness, reflecting large-scale validation and one implemented pathway rather than proven model-specific causal benefit.

#### 6.1.2 Anastomotic leakage, SSI, ileus, readmission, and recovery-related outcomes

Anastomotic leakage is a high-impact but relatively infrequent endpoint. Machine-learning models have incorporated demographic, nutritional, inflammatory, tumour-related and operative variables. Potential actions include optimisation, selective diversion, intensified monitoring and earlier diagnostic imaging. However, an AUC alone is insufficient: false-negative predictions may delay recognition of leakage, whereas false-positive predictions may lead to unnecessary diversion or intervention. Threshold-specific sensitivity, predictive values, calibration and net benefit are therefore particularly important (85–87).

SSI and postoperative ileus represent potentially modifiable but highly context-dependent endpoints. SSI models may inform nutritional optimisation, infection-prevention measures and wound monitoring, whereas ileus models may support opioid-sparing analgesia, mobilisation, fluid management and gastrointestinal monitoring. Their transportability is limited by differences in SSI definitions, enhanced-recovery protocols, operative practice and discharge criteria (88–91).

Readmission, prolonged length of stay and composite complications are also influenced by health-system factors. Models may support discharge planning and resource allocation, but their outputs depend on local bed availability, rehabilitation access, social support, coding practice and readmission thresholds. External validation should therefore include clinically different healthcare systems rather than only another random split from the same institution (92–95).

Several studies enrolled mixed benign and malignant colorectal-surgery populations. Such studies provide indirect evidence for CRC unless the cancer proportion and cancer-specific performance are reported. Cancer-specific cohorts should therefore be distinguished from general colorectal-surgery cohorts in the evidence tables. Overall, leakage-, SSI-, ileus-, readmission- and length-of-stay models remain early-stage in translational readiness. Prospective studies must determine whether prespecified model-triggered actions reduce leakage, surgical-site infection, ileus, readmission or prolonged length of stay (96).

### 6.2 Long-term survival prediction

Long-term survival models estimate overall, cancer-specific or disease-specific survival rather than a specific postoperative recurrence event. Their potential value is incremental prognostic information beyond tumour stage, site, grade, molecular subtype, treatment and comorbidity.

#### 6.2.1 WSI-based survival prediction

H&E WSIs contain information on glandular architecture, stromal composition, immune infiltration, tumour budding, necrosis and the invasive front. Kather et al. (97) demonstrated multicentre feasibility of extracting prognostic information from CRC histology, while Skrede et al. (98) developed and validated a deep-learning prognostic marker for CRC outcomes. Jiang et al. (99) subsequently reported an end-to-end system with multicentre external validation for disease-specific survival. These studies provide evidence that WSI-based models can detect prognostically relevant morphology beyond conventional visual assessment.

Their clinical role nevertheless remains uncertain. Scanner, staining and tissue-sampling variation may affect reproducibility, and few studies demonstrate that the model provides sufficient incremental value to alter adjuvant treatment or surveillance. Among the WSI studies reviewed here, no validated threshold was linked to a prespecified change in adjuvant treatment or surveillance (100).

#### 6.2.2 Clinicopathological and population-based models

Machine learning can model non-linear associations among routinely collected variables, but its incremental value depends on comparison with well-specified conventional models. Buk Cardoso et al. (101) used a large Brazilian dataset and found that stage remained the dominant predictor, followed by surgical approach, hospitalisation-related characteristics and age.

Susič et al. (102) analysed 1236 patients and 118 candidate variables to predict binary survival from 1 to 5 years. Eleven algorithms were evaluated using repeated 5-by-2-fold cross-validation. Logistic regression was the best-performing model, with an AUC of 0.850 (95% CI: 0.840–0.860) for 1-year survival and 0.872 (95% CI: 0.861–0.882) for 5-year survival when 20 variables were used. Predicted probabilities broadly agreed with Kaplan–Meier estimates across the evaluated intervals. The study compared machine-learning predictions with Kaplan–Meier and Cox methods, but did not establish that the model was clearly superior to both. In addition, some highly ranked variables, including probable recurrence within five years, require careful temporal interpretation because they may not be available at the intended prediction time. The findings should therefore be described as internally cross-validated discrimination with broad agreement with Kaplan–Meier estimates, not as proven superiority over conventional survival analysis.

Explainable models may identify subgroup-specific associations, but feature importance is not causal evidence. In a Korean nationwide cohort, stage and CEA were important across age groups, while several metabolic variables appeared more influential in younger patients. Such findings may generate hypotheses but require independent validation before influencing care (103).

#### 6.2.3 Genomic and multimodal prognostic models

Genomic and multimodal models may better represent interactions among tumour biology, treatment and host factors, but are vulnerable to missing-data bias and platform dependence. Altaf et al. (104) used AACR GENIE genomic data and XGBoost to predict overall survival in stage IV CRC. Clinical implementation would require harmonised sequencing, external validation and integration with metastatic burden, performance status and systemic-treatment exposure.

Models developed after hepatectomy for colorectal liver metastases address a distinct clinical population and should not be generalised to all CRC. They may support post-hepatectomy counselling, but must be interpreted according to metastatic burden, resectability and treatment history (105).

Long-term survival prediction was classified as early-stage in translational readiness. WSI models have comparatively stronger multicentre validation, but most survival models have not demonstrated incremental net benefit, a defined treatment pathway or improved outcomes. No validated score threshold has been linked to changes in adjuvant treatment or surveillance intensity (106).

### 6.3 Recurrence prediction

Recurrence prediction concerns disease arising after curative-intent treatment, including local or regional recurrence, metachronous distant metastasis and time to recurrence. It is distinct from the synchronous-metastasis assessment discussed in Section 3.4. DFS and RFS should not automatically be treated as pure recurrence endpoints because their definitions may include death or a new primary malignancy.

MRI radiomics has been widely studied in rectal cancer. In 600 patients with negative pretreatment CEA from three centres, Fu et al. (107) developed and evaluated an All-Clin+Rad model for DFS. C-index values were 0.775 (95% CI: 0.723–0.827) in the development cohort, 0.739 (95% CI: 0.654–0.823) in the internal test cohort and 0.822 (95% CI: 0.720–0.924) in the external test cohort. These values should not be summarised as approximately 0.81–0.82 across all cohorts. By contrast, the early-onset rectal cancer model reported by Xie et al. (108) developed a prediction model for 264 early-onset rectal cancer (EORC) patients. The EORC-ClinPathRadiom model, integrating clinicopathological characteristics and MRI-based radiomics features, achieved C-index values of 0.82 (95% CI: 0.75–0.88) in the training set, 0.82 (95% CI: 0.74–0.91) in the internal test set, and 0.81 (95% CI: 0.63–0.99) in the external test set, effectively stratifying patients into high-risk and low-risk recurrence groups (HRs: 8.96, 6.81, and 7.46, respectively).

Other rectal MRI studies addressed more specific endpoints. Sluckin et al. (109) analysed 196 patients from three Dutch tertiary centres and used primary and restaging MRI with clinical features to predict locoregional and lateral locoregional recurrence, with AUCs of 0.78 (95% CI: 0.60-0.96) and 0.80 (95% CI: 0.49-1.0). This was a retrospective multicentre study, not a single-centre internal evaluation. Liu et al. (110) enrolled 195 patients from two centres and reported development and external validation of an RS-EPI DWI model. The merged model achieved high predictive performance for recurrence or metastasis (ROM) with 1-, 3-, and 5-year AUCs of 0.887 (95% CI: 0.816–0.958), 0.813 (95% CI: 0.550–0.830), and 0.794 (95% CI: 0.655–0.932) in the training cohort, and 0.819 (95% CI: 0.673–0.965), 0.795 (95% CI: 0.599–0.991), and 0.783 (95% CI: 0.580–0.987) in the external validation cohort, respectively.

Models predicting metachronous liver metastasis after treatment are also recurrence models. Lee et al. (111) developed a prediction model for 5-year metachronous liver metastasis (5YLM) in 2,019 patients with stage I–III colorectal cancer using preoperative abdominal CT. The study employed a convolutional neural network (VGG16) to extract liver imaging features, which were reduced to principal components (PCs). The logistic-regression model combining PC1 with clinical variables achieved a mean test-set AUC of 0.747 ± 0.036, compared with 0.709 ± 0.038 for clinical variables alone (mean ± SD across 100 repeated train/test iterations). The original study did not report an inferential comparison supporting a statistically significant difference between the two models. Taghavi et al. (112) conducted a multicentre study analysing 91 colorectal cancer patients to predict metachronous liver metastases using CT radiomics. The radiomics model and the combined model (radiomics + clinical features) both achieved an AUC of 86% (95% CI: 85–87%) in the validation cohort, significantly outperforming the clinical model alone (AUC = 71%; 95% CI: 69–72%). Li et al. (113) developed a fusion model from primary-tumour CT radiomics and clinical variables. Although the imaging was obtained before surgery, the predicted outcome occurred after definitive treatment and is therefore relevant to postoperative surveillance rather than baseline synchronous staging.

Multimodal and biomarker approaches remain exploratory. Yao et al. (114) developed deep learning-based multiomics models for predicting postoperative distant metastasis (DM) and 3-year disease-free survival (DFS) in 521 colorectal cancer patients across two centres. In the training set, the deep learning radiomics (DLRS) and deep learning pathomics (DLPS) models achieved AUCs of 0.931 (95% CI: 0.898–0.965) and 0.914 (95% CI: 0.873–0.954), respectively, while the integrated Nomogram 1 (radiological + DLRS) and Nomogram 2 (pathological + DLPS) models achieved AUCs of 0.938 (95% CI: 0.907–0.969) and 0.930 (95% CI: 0.898–0.963). Serial CEA models may be more readily integrated into surveillance, although timing, assay variation, smoking, inflammation and recurrence site must be considered. Evidence for NLP-based recurrence prediction remains insufficient; extraction of follow-up variables from free text should not be presented as validated prediction.

Recurrence after colorectal liver-metastasis resection is a separate high-risk setting. Fu et al. (115) analysed 282 patients from three centres and included training, internal-validation and external-validation cohorts, with calibration and decision-curve analyses. This design therefore provides geographically external validation. Other CRLM models have combined clinical, morphological and molecular variables, but they should not be generalised to stage I–III primary CRC (116, 117).

Recurrence prediction remains early-stage in translational readiness. External validation of discrimination is increasing, but no model has shown that model-guided surveillance improves efficiency, time to salvage treatment or patient outcomes. Their immediate role is research stratification; model-guided surveillance has not been shown to improve detection of salvageable recurrence or patient outcomes. Representative studies are summarised in **Table 6**.

Across perioperative risk, survival and recurrence prediction, current evidence does not consistently establish incremental clinical value over established clinical or clinicopathological models. Large registry-derived mortality models achieved strong discrimination, but most were not compared with established risk tools using external calibration and decision-curve analysis; moreover, the Danish implementation study evaluated an AI-informed multicomponent pathway, so its reported benefit cannot be attributed to the prediction model alone (82–84). For long-term prognosis, stage remained the dominant predictor in population-based data (101), while Susič et al. found that conventional logistic regression performed best among 11 evaluated algorithms without demonstrating clear superiority over conventional survival methods (102). Imaging and multimodal data may provide additional information, but reported gains are often modest or supported only by retrospective validation. For example, adding liver CT features to clinical variables increased the AUC for metachronous liver-metastasis prediction from 0.709 to 0.747 (111), whereas higher performance reported by several radiomics or multimodal models has not been linked to changes in adjuvant treatment, surveillance intensity, salvageable recurrence detection or survival. Thus, higher AUC or C-index values alone should not be interpreted as clinically meaningful improvement. Incremental value should be demonstrated by comparing a locked AI model with a prespecified clinical baseline in the same representative external cohort and by reporting changes in discrimination together with calibration, threshold-specific predictive values and decision-curve net benefit. Prospective impact studies should then determine whether model-guided actions improve management or patient outcomes without increasing unnecessary intervention.

## 7 Clinical translation, limitations, and future directions

### 7.1 Availability, validation, and clinical-impact evidence

Model validation, workflow evaluation and clinical-impact evaluation represent different stages of evidence. Internal or external validation estimates predictive performance in new data. Prospective validation evaluates performance in prospectively collected patients but does not constitute clinical impact if predictions do not guide management. Reader-assistance studies determine whether AI changes clinician performance, whereas clinical-impact studies require the model to be incorporated into care and assess resulting decisions, harms, resource use or patient outcomes. These categories are evaluated separately in the task-level evidence matrix and summarised visually in **Figure 1**, whereas **Table 7** focuses specifically on reported model availability and implementation status. Across the 17 clinical tasks, 16 had no prospective model-impact evidence. The only partial exception was a non-randomised evaluation of an AI-informed multicomponent perioperative pathway, in which the effect of the prediction model could not be separated from the accompanying care interventions. Thus, no task currently has prospective model-specific evidence that AI-guided care improves clinical decisions or patient outcomes. Decision-curve analysis was reported only for selected models and represents estimated net benefit under specified assumptions rather than demonstrated benefit in clinical practice. This lack of prospective clinical-impact evidence remains a principal barrier to clinical translation.

**Figure 1:**
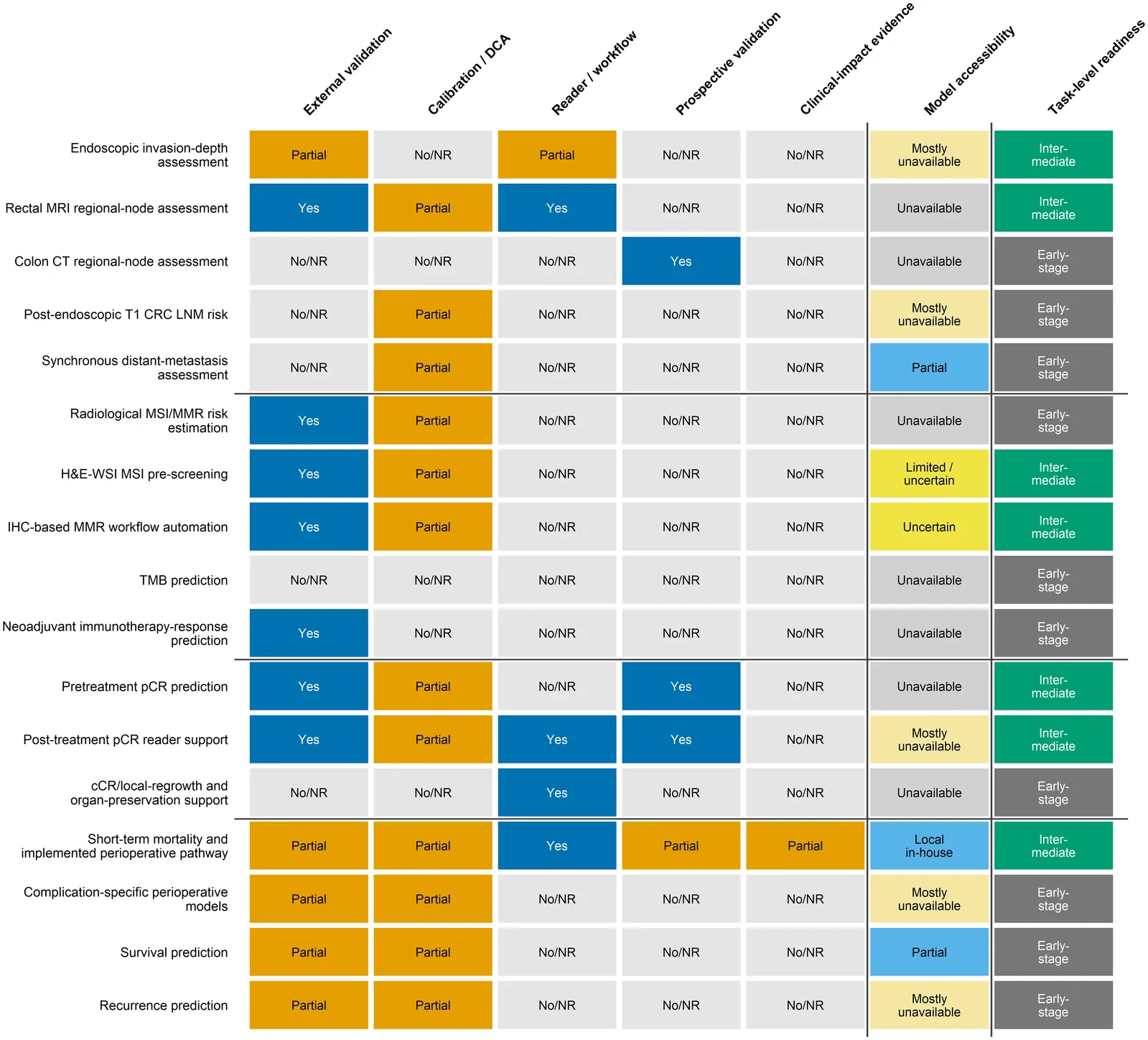
Clinical-readiness heatmap of artificial intelligence applications in colorectal cancer. Readiness was assessed according to external validation, calibration or decision-curve analysis, reader/workflow evaluation, prospective validation, clinical-impact evidence, and model accessibility. Blue, orange, and grey indicate “Yes,” “Partial,” and “No/NR,” respectively; green and dark grey indicate intermediate and early-stage readiness. DCA, decision-curve analysis; NR, not reported; MSI, microsatellite instability; MMR, mismatch repair; pCR, pathological complete response; cCR, clinical complete response; TMB, tumour mutational burden.

**Table 7:** Reported availability and implementation status of selected artificial-intelligence applications in colorectal cancer.

Application area	Availability reported in the cited publication	Representative evidence	Independent current verification	Implementation interpretation	Key caveats
MSI/MMR pre-screening from H&E WSIs	Clinically oriented, independently validated pre-screening model	MSIntuit independent blinded validation (54)	Routine deployment/commercial availability was not independently verified	High-sensitivity triage only; confirmatory IHC, PCR or NGS remains required.	Scanner, staining, workflow, cost-effectiveness, regulatory status and maintenance require independent evaluation.
Perioperative risk decision support	Implemented locally as an in-house AI-enabled perioperative pathway	Danish implementation study (84)	Implemented in the reporting centre; transportability elsewhere unverified	A real-world workflow example, but the evaluated intervention was AI prediction plus an individualised treatment bundle.	Non-randomised before-and-after design; outcome differences cannot be attributed to the prediction model alone.
Postoperative lower-limb DVT risk	Web calculator reported in the original publication	Random-forest model with external validation (118)	Current accessibility, maintenance and governance not independently verified	Research-derived interface; prospective workflow and safety evaluation required.	Publication of a calculator does not establish current access, regulatory approval or clinical deployment.
CRC survival prediction	Web calculators reported in the original publication	Early- and late-onset CRC survival tools (119)	Current accessibility, maintenance and governance not independently verified	Exploratory risk communication only; should not replace guideline-based prognostic assessment.	Calibration across treatment eras and health systems and prospective impact are unproven.
Radiomics/pathomics for LNM, metastasis, pCR, TMB, recurrence and survival	Mostly research-only	Representative models across these task groups (29–43, 56–58, 61–75, 97–117)	Availability varies; no general deployment assumption was made, and reported open-source exceptions were recorded explicitly.	Require model/code access, local calibration, usability, cybersecurity, monitoring and prospective impact evaluation.	Nomograms or favourable AUCs do not establish deployability.
IHC-based MMR workflow automation	Single-cell AI assessment of MMR-protein IHC reported in two trial cohorts	AIMMeR (55)	Independent routine deployment and maintenance not verified	Potential automation of MMR-protein expression assessment; not H&E-based MSI inference.	Requires pathology-workflow validation, governance, cost evaluation and confirmation against the intended biological reference standard.
Synchronous distant-metastasis radiomics	Custom Python analysis code reported as public	Jiang et al. (41)	Repository was reported by the article; independent reproducibility, trained weights and maintenance not verified	Code availability improves transparency for a single-centre internally tested model.	Public analysis code does not establish an externally validated or deployable clinical system.
WSI-based CRC survival prediction	Source code and trained model weights reported as public for one multicentre model	Jiang et al. (99)	Reported open-source package; current maintenance and cross-platform deployment not independently verified	A reproducible exception to the general pattern of unavailable research models.	Open code and weights do not demonstrate incremental clinical utility or regulatory readiness.

CRC, colorectal cancer; H&E, haematoxylin and eosin; IHC, immunohistochemistry; MMR/MMRd, mismatch repair/deficiency; MSI, microsatellite instability; PCR, polymerase chain reaction; WSI, whole-slide image. Reported availability is distinguished from independently verified maintenance, regulatory status and deployment. NR, not reported.

MSIntuit represents an independently validated, clinically oriented example of H&E-based MSI pre-screening. In a blinded cohort of 600 consecutive patients, sensitivity was approximately 0.96–0.98 and specificity approximately 0.46–0.47. This profile supports high-sensitivity triage for confirmatory testing rather than definitive MSI diagnosis. Independent validation does not by itself establish routine availability, regulatory approval, cost-effectiveness or cross-platform deployment (54).

Reported web calculators also require cautious wording. A postoperative lower-limb DVT calculator achieved AUCs of 0.942, 0.897 and 0.891 in training, testing and external-validation cohorts, and another study reported web-based survival tools for early- and late-onset CRC. Publication of a web interface does not demonstrate that it remains accessible, maintained, secure, independently tested or integrated into care. Such tools should be described as reported research-derived web calculators unless their current operational status has been verified (118, 119).

Most radiomics, pathomics, deep-learning and multimodal models reviewed are not available as independently reproducible clinical products. Code, weights, preprocessing specifications, input definitions, software requirements, regulatory status and maintenance plans are frequently unavailable. A nomogram or web page should not be equated with deployable software. The implementation status of selected examples is summarised in **Table 7**.

### 7.2 Methodological limitations and research priorities

For treatment-response prediction specifically, selected MRI and radiopathomic models have maintained discrimination in independent or prospective multicentre cohorts (61–63), supporting partial cross-institutional reproducibility. However, protocol-independent generalisability remains unproven because performance has rarely been stratified by scanner characteristics, acquisition protocols, segmentation and preprocessing workflows, or treatment regimens, and cross-site calibration and fixed-threshold transportability have seldom been evaluated (69, 70). Future validation should therefore use locked analytical pipelines and prespecified protocol- and regimen-stratified analyses, with calibration and clinically relevant thresholds assessed across institutions.

Discrimination should be accompanied by calibration, clinically interpretable thresholds and predictive values. Decision-curve analysis is needed when a prediction could change treatment, surgery, surveillance or resource allocation. Time-to-event models should account for censoring and competing risks, and low-frequency complications require explicit handling of class imbalance. Comparisons should use established clinical, radiological or pathological standards rather than weak baselines.

Terminology should distinguish conventional regression, radiomics with statistical modelling, machine learning and deep learning. Use of penalised logistic regression or a radiomics score does not automatically indicate an advanced AI system. The manuscript should describe the algorithm and data-processing pipeline precisely rather than relying on the generic label “AI”.

These methodological categories are overlapping rather than mutually exclusive: radiomics is a feature-engineering pipeline that may be coupled with regression, conventional machine learning or deep learning, whereas multimodal AI refers to the integration of different data sources rather than a specific algorithm. Conventional machine learning is well suited to structured clinicopathological variables or predefined radiomic features and may offer relatively transparent variable-level interpretation, but its performance depends heavily on feature selection and may not exceed that of a well-specified regression model (36, 102). Deep learning can learn complex spatial representations directly from endoscopic images, radiological examinations or WSIs and may reduce manual feature engineering, but it generally requires representative datasets and is vulnerable to data leakage, limited interpretability and performance degradation across centres, scanners or populations (29, 34, 37). Radiomics can extract quantitative information from routinely acquired imaging and may be more feasible than end-to-end deep learning in modest datasets, but segmentation, acquisition, reconstruction and feature-standardisation differences limit reproducibility (4, 120–124). Multimodal models may improve prediction by combining complementary imaging, pathology, molecular and clinical information (41, 57, 62, 63), but they introduce missing-data, temporal-alignment, harmonisation and workflow burdens, and their incremental value should be confirmed through ablation analysis and external validation. Accordingly, no approach is inherently superior; clinical value depends on the intended decision, data quality, transportability, calibration, accessibility and workflow integration.

Tumour heterogeneity represents an additional limitation for AI-based molecular-biomarker inference. Intratumoural spatial heterogeneity in clonal composition, glandular morphology, immune infiltration, stromal response, necrosis and metabolism means that a single biopsy, whole-slide image, imaging slice or selected region of interest may not represent the entire tumour. Tumours sharing MSI-H/dMMR or TMB-high status may therefore display different imaging or histological phenotypes, whereas biologically unrelated processes may generate similar features. Intertumoural heterogeneity across colonic and rectal sites, disease stages, molecular subtypes and patient populations may also encourage models to learn cohort-specific surrogate features rather than stable biomarker-related biology. Temporal evolution after treatment and differences between primary and metastatic lesions may further impair transportability when the specimen or time point used for validation differs from that used for model development. These effects may reduce within-patient repeatability and the stability of discrimination, calibration and clinical thresholds across settings (47, 52). Future studies should incorporate patient-level splitting, multiregional or multiblock sampling, whole-tumour and subregion analyses, and independent multicentre validation stratified by tumour site, stage, specimen type and treatment status. Longitudinal or paired primary–metastatic evaluation, uncertainty reporting and predefined abstention pathways are also needed. Clinically consequential or discordant molecular predictions should undergo confirmatory IHC, PCR or NGS.

Explainability represents a distinct translational requirement from predictive discrimination. In structured clinicopathological or radiomics models, regression coefficients, global feature importance and local methods such as SHapley Additive exPlanations (SHAP) may indicate which variables contribute to population-level or individual predictions. In deep-learning models using radiological images or whole-slide pathology images, saliency maps, attention maps, occlusion analysis and related localisation approaches may help determine whether the model focuses on the primary tumour, invasive front, lymph nodes or relevant immune–stromal regions rather than scanner, staining or background artefacts (60, 72, 75, 103, 109, 117). Such outputs may support clinician review, error analysis, identification of shortcut learning and recognition of out-of-distribution cases. However, *post-hoc* explanations may be unstable across preprocessing procedures, institutions and model updates and may not faithfully represent the model’s decision process. Clinically plausible attention maps or feature rankings should therefore not be interpreted as biological validation, causal evidence or proof of clinical utility. Translation-oriented studies should prespecify the intended user and decision, evaluate explanation fidelity and stability, compare localisation outputs with expert annotations where appropriate, report consistency across patient subgroups and assess whether explanations improve clinician accuracy and confidence calibration without increasing automation bias. XAI should complement, rather than replace, independent external validation, calibration, decision-curve analysis and prospective human–AI interaction or clinical-impact evaluation.

Reporting guidance should be matched to the stage and design of AI evaluation rather than cited collectively. TRIPOD+AI, the current designation of the updated TRIPOD guidance, applies to the development, validation and updating of diagnostic and prognostic prediction models using either regression or machine-learning methods. It requires transparent reporting of the intended use, data sources, participant selection, predictors and outcomes, missing-data handling, model specification, performance, subgroup assessment and model availability (8). PROBAST+AI complements TRIPOD+AI by supporting assessment of study quality, risk of bias and applicability (9). Modality-specific guidance also remains relevant: CLAIM applies to medical-imaging AI, whereas CLEAR and IBSI address radiomics reporting, preprocessing and feature standardisation (121–124).

Prospective research should progress from silent validation to reader-assistance and workflow studies, followed by clinical-impact evaluation where justified. DECIDE-AI is particularly relevant to early live clinical evaluation, including actual clinical performance, safety, usability, human factors and workflow integration (10). When an AI system is evaluated as a clinical intervention, SPIRIT-AI should guide the prespecified trial protocol and CONSORT-AI the completed trial report, together with the applicable core SPIRIT and CONSORT guidance (126, 127). These extensions require transparent specification of the intended users and clinical pathway, algorithm version, input acquisition and preprocessing, handling of poor-quality or unavailable inputs, model outputs and action thresholds, required user expertise, human–AI interaction and performance-error analysis. Adherence would improve reproducibility and clarify whether benefit arises from the model itself or from the wider human–AI workflow. However, reporting compliance does not itself demonstrate low risk of bias or clinical utility. Future CRC trials should prospectively register a locked or version-controlled model, use clinically relevant comparators and outcomes, evaluate subgroup performance and harms, and prespecify failure, update and model-drift monitoring.

Fairness and uncertainty are essential when predictions could influence surgery or treatment intensity. Feature attribution and attention maps may assist error analysis but do not demonstrate causal relationships. Studies should report subgroup performance, uncertainty, out-of-distribution behaviour and failure pathways, with post-deployment monitoring, recalibration, version control and clinical oversight.

This review has several limitations. It is a structured narrative review rather than a systematic review, is restricted to English-language publications, and does not include a formal study-level risk-of-bias assessment. Representative studies were prioritised according to validation design and clinical relevance, so selection bias cannot be excluded. Model availability and implementation status may also change after the final search date. The task-level evidence matrix and translational-readiness assignments are presented in **Figure 1**.

### 7.3 Emerging technologies for next-generation CRC decision support

Next-generation CRC decision-support systems may move from isolated, task-specific predictors towards multimodal foundation-model architectures that integrate endoscopic images, CT or MRI, H&E whole-slide images, molecular data, structured clinicopathological variables and clinical text. In contrast to conventional multimodal models developed for a single endpoint, pretrained modality-specific or multimodal encoders may be adapted across staging, molecular pre-screening, treatment-response, recurrence and perioperative-risk tasks while reducing dependence on large task-specific labelled datasets. A recent foundation-model-enabled framework integrated pathology, CT and clinical reports from 1,679 patients across four centres and explicitly addressed incomplete modalities (128). Although this study provides early evidence for multimodal prognostic integration, it remains a retrospective task-specific model rather than a general-purpose clinical foundation model. Missing-not-at-random modalities, temporal misalignment, modality dominance, inherited pretraining bias, computational requirements and model-version changes may all impair calibration and transportability. Prospective studies should therefore compare multimodal systems with strong unimodal and clinical baselines, conduct ablation analyses, and report performance for each pattern of modality availability (41, 57, 62, 63, 74, 128).

Federated learning may complement these architectures by enabling institutions to train shared models without centralising raw patient data. In 1,305 patients from two rectal-cancer centres, a federated model predicted near-complete pathological response with an AUC of 0.77, whereas models trained at one centre transferred poorly to the other centre, with AUCs of 0.50 and 0.59 (129). In another three-centre study of 2,959 patients undergoing colorectal surgery, federated prediction of 90-day mortality achieved performance similar to centralised training, although differential-privacy measures reduced predictive performance (130). Swarm learning has likewise enabled decentralised training on CRC whole-slide images from more than 5,000 patients for MSI/dMMR and BRAF prediction, with performance approaching that of models trained on pooled data (131). These findings demonstrate technical feasibility but do not mean that federated learning automatically guarantees privacy, fairness or generalisability. Model updates may remain vulnerable to information leakage, and non-identically distributed populations, scanners, treatment pathways, endpoint definitions and missingness patterns may create unequal performance across participating centres. Future federated studies should therefore incorporate secure aggregation, appropriately calibrated differential privacy, harmonised reference standards, site-level calibration and fairness assessment, and independent external or prospective validation.

Explainable AI (XAI) may provide the human-oversight layer of these systems by linking predictions to clinically reviewable imaging regions, histological patterns and patient variables. Saliency or attention maps, feature attribution, counterfactual explanations and calibrated uncertainty could help clinicians identify shortcut learning, review discordant cases, recognise out-of-distribution inputs and determine when a model should abstain rather than issue a potentially unsafe recommendation (72, 103, 109, 117). However, a visually plausible heatmap or feature ranking does not establish explanation fidelity, causal relevance or clinical utility. Explanations may vary across preprocessing pipelines, institutions and model versions and may themselves increase automation bias. Translation-oriented studies should therefore evaluate explanation fidelity and stability, agreement with expert annotations, subgroup consistency and whether explanations improve clinician accuracy and confidence calibration in prospective human–AI interaction studies. Multimodal foundation models, federated learning and XAI are consequently complementary rather than sufficient technologies: they may improve representation learning, access to diverse multicentre data and model auditability, respectively, but next-generation CRC systems must still be decision-specific, externally validated, calibrated, version-controlled and prospectively evaluated for safety, workflow effects and patient outcomes.

## 8 Conclusion

The central question for colorectal cancer AI is no longer whether high retrospective discrimination can be achieved, but whether models learn stable and clinically meaningful signals that remain valid across tumour sites, disease stages, specimen and imaging protocols, treatment pathways and healthcare systems. It remains unresolved whether apparent performance gains reflect reproducible tumour biology or cohort-specific shortcuts; whether AI provides calibrated, incremental benefit beyond expert assessment, TNM staging and established molecular testing; and whether model outputs can be linked to prespecified clinical actions at thresholds that reflect the asymmetric consequences of missed disease and unnecessary treatment. Models must also communicate uncertainty and support safe abstention, confirmatory testing and escalation when inputs fall outside their validated scope.

Research should therefore begin with a clearly defined clinical decision, intended user, input time point, reference standard, comparator, action threshold and consequence of error. Priorities include temporal and geographical validation of locked models in representative multicentre cohorts; rigorous patient-level data separation; assessment of calibration, clinical utility, subgroup performance, fairness, uncertainty and out-of-distribution failure; and reproducible reporting of data provenance, model versions and evaluation procedures. Colorectal cancer heterogeneity further requires multi-region, multiblock or whole-tumour sampling and longitudinal validation across primary tumours, metastases and treatment time points. Multimodal foundation models, federated learning and explainable AI may address fragmented information, limited data sharing and interpretability, respectively, but their novelty alone should not be considered evidence of clinical readiness.

Promising systems should progress from retrospective and silent prospective validation to human–AI workflow studies and, ultimately, comparative or randomised clinical-impact evaluations. These studies should measure changes in management, missed disease, unnecessary interventions, treatment timeliness, complications, organ preservation, salvageability, patient-reported outcomes and cost-effectiveness rather than discrimination metrics alone. Deployment should additionally include monitoring for data and performance drift, recalibration procedures, version control, auditability and clear human accountability. Among the representative studies reviewed across 17 clinical tasks, 16 had no reported prospective model-impact evidence; the only partial exception was a non-randomised multicomponent perioperative pathway in which the independent effect of the prediction model could not be isolated. Accordingly, the absence of prospective model-specific clinical-impact evidence is a central conclusion of this review. The immediate research priority is prospective evaluation of predefined model-guided actions, harms, workflow effects and patient outcomes.
