# Supplementary material for: Artificial intelligence and computational prediction models for risk stratification, treatment response, and outcomes in colorectal cancer: a narrative review
Source: Front Oncol. 2026 Sep 22;16:1944198. doi: 10.3389/fonc.2026.1944198 (PMC13639575; doi:10.3389/fonc.2026.1944198)
Supplement: Supplementary file 1 [file Table1.doc]

**Search strategies**

**PubMed/MEDLINE**

("Colorectal Neoplasms"[Mesh]

OR "colorectal cancer"[Title/Abstract]

OR "colorectal cancers"[Title/Abstract]

OR "colorectal neoplasm"[Title/Abstract]

OR "colorectal neoplasms"[Title/Abstract]

OR "colorectal tumor"[Title/Abstract]

OR "colorectal tumors"[Title/Abstract]

OR "colorectal tumour"[Title/Abstract]

OR "colorectal tumours"[Title/Abstract]

OR "colon cancer"[Title/Abstract]

OR "colonic cancer"[Title/Abstract]

OR "rectal cancer"[Title/Abstract])

AND

("Artificial Intelligence"[Mesh]

OR "Machine Learning"[Mesh]

OR "Deep Learning"[Mesh]

OR "Neural Networks, Computer"[Mesh]

OR "artificial intelligence"[Title/Abstract]

OR "machine learning"[Title/Abstract]

OR "deep learning"[Title/Abstract]

OR radiomic*[Title/Abstract]

OR pathomic*[Title/Abstract]

OR "digital pathology"[Title/Abstract]

OR "whole-slide imaging"[Title/Abstract]

OR "whole slide imaging"[Title/Abstract]

OR "whole-slide image"[Title/Abstract]

OR "whole slide image"[Title/Abstract]

OR "convolutional neural network"[Title/Abstract]

OR "convolutional neural networks"[Title/Abstract]

OR transformer*[Title/Abstract]

OR "natural language processing"[Title/Abstract]

OR "computer-aided diagnosis"[Title/Abstract]

OR "computer-assisted diagnosis"[Title/Abstract]

OR "multimodal model"[Title/Abstract]

OR "multimodal models"[Title/Abstract]

OR "multimodal prediction"[Title/Abstract])

AND

(predict*[Title/Abstract]

OR prognos*[Title/Abstract]

OR "risk stratification"[Title/Abstract]

OR staging[Title/Abstract]

OR endoscop*[Title/Abstract]

OR colonoscop*[Title/Abstract]

OR "invasion depth"[Title/Abstract]

OR "depth of invasion"[Title/Abstract]

OR "submucosal invasion"[Title/Abstract]

OR "endoscopic resection"[Title/Abstract]

OR "endoscopic treatment"[Title/Abstract]

OR "T1 colorectal cancer"[Title/Abstract]

OR "lymph node metastasis"[Title/Abstract]

OR "lymph-node metastasis"[Title/Abstract]

OR "nodal staging"[Title/Abstract]

OR "lateral lymph node"[Title/Abstract]

OR metastas*[Title/Abstract]

OR "synchronous metastasis"[Title/Abstract]

OR "metachronous metastasis"[Title/Abstract]

OR "liver metastasis"[Title/Abstract]

OR "lung metastasis"[Title/Abstract]

OR "bone metastasis"[Title/Abstract]

OR "microsatellite instability"[Title/Abstract]

OR "mismatch repair"[Title/Abstract]

OR MSI[Title/Abstract]

OR dMMR[Title/Abstract]

OR pMMR[Title/Abstract]

OR "tumor mutational burden"[Title/Abstract]

OR "tumour mutational burden"[Title/Abstract]

OR TMB[Title/Abstract]

OR immunotherap*[Title/Abstract]

OR neoadjuvant[Title/Abstract]

OR chemoradiotherap*[Title/Abstract]

OR "total neoadjuvant therapy"[Title/Abstract]

OR "treatment response"[Title/Abstract]

OR "pathological complete response"[Title/Abstract]

OR "pathologic complete response"[Title/Abstract]

OR pCR[Title/Abstract]

OR "clinical complete response"[Title/Abstract]

OR cCR[Title/Abstract]

OR "organ preservation"[Title/Abstract]

OR "organ-preservation"[Title/Abstract]

OR "watch and wait"[Title/Abstract]

OR "watch-and-wait"[Title/Abstract]

OR perioperative[Title/Abstract]

OR postoperative[Title/Abstract]

OR "postoperative complication"[Title/Abstract]

OR "postoperative complications"[Title/Abstract]

OR "anastomotic leak"[Title/Abstract]

OR "anastomotic leakage"[Title/Abstract]

OR "surgical site infection"[Title/Abstract]

OR "surgical-site infection"[Title/Abstract]

OR SSI[Title/Abstract]

OR "postoperative ileus"[Title/Abstract]

OR readmission[Title/Abstract]

OR "length of stay"[Title/Abstract]

OR "deep vein thrombosis"[Title/Abstract]

OR DVT[Title/Abstract]

OR "venous thromboembolism"[Title/Abstract]

OR VTE[Title/Abstract]

OR mortality[Title/Abstract]

OR survival[Title/Abstract]

OR recurrence[Title/Abstract]

OR relapse[Title/Abstract])

AND ("2010/01/01"[Date - Publication] : "2026/05/31"[Date - Publication])

AND English[Language]

**Web of Science Core Collection**

TS=("colorectal cancer" OR "colorectal neoplasm*" OR "colorectal tumor*" OR "colorectal tumour*" OR "colon cancer" OR "colonic cancer" OR "rectal cancer") AND TS=("artificial intelligence" OR "machine learning" OR "deep learning" OR radiomic* OR pathomic* OR "digital pathology" OR "whole-slide imag*" OR "whole slide imag*" OR "convolutional neural network*" OR transformer* OR "natural language processing" OR "computer-aided diagnosis" OR "computer-assisted diagnosis" OR "multimodal model*" OR "multimodal prediction") AND TS=(predict* OR prognos* OR "risk stratification" OR staging OR endoscop* OR colonoscop* OR "invasion depth" OR "depth of invasion" OR "submucosal invasion" OR "endoscopic resection" OR "endoscopic treatment" OR "T1 colorectal cancer" OR "lymph node metastas*" OR "nodal staging" OR "lateral lymph node*" OR metastas* OR "synchronous metastasis" OR "metachronous metastasis" OR "liver metastasis" OR "lung metastasis" OR "bone metastasis" OR "microsatellite instability" OR "mismatch repair" OR MSI OR dMMR OR pMMR OR "tumor mutational burden" OR "tumour mutational burden" OR TMB OR immunotherap* OR neoadjuvant OR chemoradiotherap* OR "total neoadjuvant therapy" OR "treatment response" OR "pathological complete response" OR "pathologic complete response" OR pCR OR "clinical complete response" OR cCR OR "organ preservation" OR "watch and wait" OR perioperative OR postoperative OR "anastomotic leak*" OR "surgical site infection*" OR "postoperative ileus" OR readmission OR "length of stay" OR "deep vein thrombosis" OR "venous thromboembolism" OR mortality OR survival OR recurrence OR relapse)

**Google Scholar**

1. "colorectal cancer" ("artificial intelligence" OR "machine learning" OR "deep learning") (endoscopy OR "invasion depth" OR "submucosal invasion" OR "endoscopic resection") 2010..2026
2. ("colorectal cancer" OR "rectal cancer" OR "colon cancer") ("machine learning" OR "deep learning" OR radiomics) ("lymph node metastasis" OR "nodal staging" OR "lateral lymph node") 2010..2026
3. ("colorectal cancer" OR "rectal cancer") ("artificial intelligence" OR radiomics OR "deep learning") ("synchronous metastasis" OR "distant metastasis" OR "liver metastasis" OR "lung metastasis" OR "bone metastasis") 2010..2026
4. "colorectal cancer" ("artificial intelligence" OR "machine learning" OR radiomics OR pathomics) ("microsatellite instability" OR "mismatch repair" OR MSI OR "tumor mutational burden" OR TMB OR immunotherapy) 2010..2026
5. "rectal cancer" ("artificial intelligence" OR "machine learning" OR "deep learning" OR radiomics OR pathomics) (neoadjuvant OR chemoradiotherapy OR "pathological complete response" OR "clinical complete response" OR "organ preservation" OR "watch and wait") 2010..2026
6. ("colorectal cancer surgery" OR "rectal cancer surgery" OR "colon cancer surgery") ("machine learning" OR "artificial intelligence") ("anastomotic leak" OR "surgical site infection" OR "postoperative ileus" OR readmission OR "length of stay" OR mortality OR "deep vein thrombosis" OR "venous thromboembolism") 2010..2026
7. "colorectal cancer" ("artificial intelligence" OR "machine learning" OR "deep learning" OR radiomics OR pathomics) (survival OR prognosis OR recurrence OR relapse OR "metachronous metastasis") 2010..2026

**ScienceDirect supplementary search**

Common concept block

("colorectal cancer" OR "colon cancer" OR "rectal cancer")

AND

("artificial intelligence" OR "machine learning" OR "deep learning"

OR radiomics OR pathomics OR "digital pathology" OR "whole-slide imaging")

Task-specific blocks run separately with the common block

1. (endoscopy OR colonoscopy OR "invasion depth" OR "submucosal invasion"

OR "endoscopic resection" OR "endoscopic treatment")

2. ("lymph node metastasis" OR "nodal staging" OR "lateral lymph node")

3. ("synchronous metastasis" OR "distant metastasis" OR "liver metastasis"

OR "lung metastasis" OR "bone metastasis")

4. ("microsatellite instability" OR "mismatch repair" OR MSI OR dMMR

OR "tumor mutational burden" OR TMB OR immunotherapy)

5. (neoadjuvant OR chemoradiotherapy OR "total neoadjuvant therapy"

OR "pathological complete response" OR "clinical complete response"

OR "organ preservation" OR "watch and wait")

6. ("postoperative complication" OR "anastomotic leak" OR "surgical site infection"

OR "postoperative ileus" OR readmission OR "length of stay" OR mortality

OR "deep vein thrombosis" OR "venous thromboembolism")

7. (survival OR prognosis OR recurrence OR relapse OR "metachronous metastasis")
